# Molecular insight into isoform specific inhibition of PI3K-α and PKC-η with dietary agents through an ensemble pharmacophore and docking studies

**DOI:** 10.1038/s41598-021-90287-3

**Published:** 2021-06-09

**Authors:** Baki Vijaya Bhaskar, Aluru Rammohan, Tirumalasetty Munichandra Babu, Gui Yu Zheng, Weibin Chen, Wudayagiri Rajendra, Grigory V. Zyryanov, Wei Gu

**Affiliations:** 1 Department of Pathophysiology, The Key Immunopathology Laboratory of Guangdong Province, Shantou University Medical College, Xinling Road, Shantou, 515041 Guangdong China; 2 Department of Organic and Biomolecular Chemistry, Ural Federal University, Ekaterinburg, 620002 Russia; 3 Department of Physiology, Shantou University Medical College, Shantou, 515031 Guangdong China; 4 Department of Zoology, Sri Venkateswara University, Tirupati, Andhra Pradesh 517502 India

**Keywords:** Chemotherapy, Drug discovery and development, Structure-based drug design

## Abstract

Dietary compounds play an important role in the prevention and treatment of many cancers, although their specific molecular mechanism is not yet known. In the present study, thirty dietary agents were analyzed on nine drug targets through in silico studies. However, nine dietary scaffolds, such as silibinin, flavopiridol, oleandrin, ursolic acid, α-boswellic acid, β-boswellic acid, triterpenoid, guggulsterone, and oleanolic acid potentially bound to the cavity of PI3K-α, PKC-η, H-Ras, and Ras with the highest binding energy. Particularly, the compounds silibinin and flavopiridol have been shown to have broad spectrum anticancer activity. Interestingly, flavopiridol was embedded in the pockets of PI3K-α and PKC-η as bound crystal inhibitors in two different conformations and showed significant interactions with ATP binding pocket residues. However, complex-based pharmacophore modeling achieved two vital pharmacophoric features namely, two H-bond acceptors for PI3K-α, while three are hydrophobic, one cat-donor and one H-bond donor and acceptor for PKC-η, respectively. The database screening with the ChemBridge core library explored potential hits on a valid pharmacophore query. Therefore, to optimize perspective lead compounds from the hits, which were subjected to various constraints such as docking, MM/GBVI, Lipinski rule of five, ADMET and toxicity properties. Henceforth, the top ligands were sorted out and examined for vital interactions with key residues, arguably the top three promising lead compounds for PI3K-α, while seven for PKC-η, exhibiting binding energy from − 11.5 to − 8.5 kcal mol^−1^. Therefore, these scaffolds could be helpful in the development of novel class of effective anticancer agents.

## Introduction

Dietary compounds derived from plants have shown a wide range of application in cancer drug discovery, especially the consumption of fruits and vegetables preferably non-starchy such as broccoli, cabbage, spinach, kale, cauliflower, carrots, lettuce, cucumber, tomato, leek, rutabaga, and turnip contain potent bioactive compounds and have shown potent anticancer properties by exhibiting inhibitory effect on different cancers^1^. The phytochemicals such as alkaloids, monoterpenes, organo-sulfides, carotenoids, flavonoids, phenolic acids, stilbenes, and isoflavones have a direct impact on human health by playing a role in regulating chemo-preventive and chemo-therapeutic approaches^2, 3^. Also, dietary compounds act as anticancer agents through various mechanisms such as anti-proliferative, apoptosis, antioxidant, anti-metastasis, and angiogenesis, and by regulating vital metabolic pathways such as Wnt/β-catenin, PI3K/Akt/mTOR, MAPK (p38, JNK, and Erk1/2), peroxisome proliferator activator receptor gamma (PPAR-γ), Sonic Hedgehog, EGFR/Kras/Braf, EGFR, VEGFR, IGF1-R, TGF-β/Smad2/3, STAT1-STAT3, NF-кB, Nrf2, TNF-α, interleukins, COX-2, 5-LOX, and cyclin-CDK complexes, respectively^4–7^. Given the importance of the dietary scaffolds, the National Cancer Institute (NCI) had conducted a chemo-preventive testing program for pre-clinical evaluation of over a thousand dietary agents with different combinations in in vitro and in vivo studies since 1987^8^, with over 40% of the tested compounds have been shown potent anticancer activity on many of the cancers; For example, breast, colon, prostate, and lung^9^. Among these key scaffolds, Indole 3 carbinol is a well-known potent broad-spectrum agent tested for anticancer activity on breast, colorectal, cervical, and endometrial cancers. Also, it exhibits antiestrogenic activity by altering the cytochrome P450 based estradiol metabolism^10^. Moreover, soy isoflavones, retinoids, vitamin E, organo-selenium, lycopene, perillyl-alcohol, and vitamin D1 scaffolds were shown prominent chemo-preventive profiles on prostate cancer^11, 12^, notably, lycopene successfully entered phase I clinical trials. In addition, polyphenols, curcumin, and epigallocatechin gallate (EGCG) significantly reduced breast cancer, while curcumin and tea polyphenols showed anti-inflammatory and cyclooxygenase-2 inhibitory activities^13, 14^. Resveratrol is a polyphenolic stilbene found abundantly in grape skin, which lessen the risk of cancer by targeting multiple signaling pathways; For example, the Wnt/β-catenin signaling pathway^15, 16^. Pterostilbene is an essential bioactive constituent found in blueberries that inhibits breast cancer stem cell proliferation and metastasis by modulating NF-κB/microRNA function^17^. Genistein, a soy isoflavone inhibits NF-κB by reducing the Hedgehog-GLI1 signaling pathway in both breast and prostate cancers^18^. Natural isothiocyanates were isolated from cruciferous plants, which suppress cell proliferation and some key cellular signaling pathways such as, NF-κB, and STAT3^19, 20^. Phenethyl isothiocyanate (PEITC), a naturally occurring isothiocyanate, has been shown to inhibit colorectal cancer stem cell inhibition by suppression the Wnt/β-catenin pathway, reducing clonogenicity, self-renewal capacity, and pluripotency^21^. Sulforaphane is a sulfur-rich compound that is important for targeting essential metabolic pathways such as Sonic hedgehog-GLI pathway, platelet-derived growth factor receptor-β, transcription factors, vascular endothelial growth factor and tumor size reduction in pancreatic cancer^22^. Similarly, a diallyl trisulfide (DATS) derived from garlic is an organosulfur agent that prevent colorectal cancer by blocking the Wnt/β-catenin pathways^23^. In virtue of these dietary scaffolds, which have broad-spectrum anticancer activity by targeting different signaling pathway, there is a pressing need to uncover relative drug targets to design inhibitors.

So far, various essential metabolic pathways and key factors have been accessed as a pool of potential therapeutic targets for the development of anticancer therapies^24^, for example, transcription factors^25, 26^, anti-apoptotic and pro-apoptotic proteins, protein kinases, cell cycle, and cell adhesion molecules, respectively. Among these key drug targets, phosphatidylinositol-3-kinase (PI3K) is a key target that is primarily affected by mutations in cancer, gene rearrangement, and gene amplification and is therefore an attractive drug target^27–31^. In addition, Protein kinase C (PKC) is such a key signaling molecule, the dysregulation of which causes cancer and diabetes^32^. Tumor receptor associate factor 2 (TRAF2) is a ring finger adaptor protein that plays a crucial role in the carcinogenicity, so it is another novel target but its role has not been properly established^33, 34^. Nuclear transcription factor kappa B (NF-κB) is a well-known transcription factor with broad spectrum therapeutic profiles that regulates approximately 200 genes related to immune response, cell differentiation, cell proliferation, and apoptosis^35–39^. Ras family proteins are small membrane-bound guanidine nucleotide-binding proteins that activate PI3K-α, AKT-mTOR, RAF-MERK-ERK, and RALGDS-RAL metabolic pathways and provide promising therapeutic targets^34, 40, 41^. In view of importance of the dietary agents in treatment of cancer, Saldanha and Tollefsbol strongly implemented a study to identify and design novel cancer inhibitors with chemo-preventive properties along with specific drug targets^42^. Therefore, the discovery of anticancer drugs based on the dietary scaffolds is a promising approach to prevent or inhibit cancer.

## Results

### Pocket volumetric analysis

The dietary compounds have been reported to have anticancer properties by exhibiting inhibition of an essential pathway and important factors such as transcription factors, anti-apoptotic and pro-apoptotic proteins, kinases, and adhesion molecules in cancer pathogenesis. Therefore, the drug targets such as PKC-η, HRas-P21, AKT-1, Ras, PI3K-α, MEKK3, NFκB-P52, MEKK2b, and TRAF2 were retrieved from the macromolecular database and examined for pocket volume, size, and shape (Fig. S2; Table S2). The volumetric evaluation explained that the PI3K-α has a cohesive binding cavity by displaying a cavity volume of 2736.6 Å as with a surface area of 2703 Å. The TRAF2 showed cavity volume of 1051 Å and the surface area of 662.5 Å. Likewise, the PKC-η revealed a cavity volume of 910.5 Å and a surface area of 843.6 Å. Similar binding pocket volumes 609.2 Å and 602.9 Å were shown by The MEKK2b and the AKT-1, while the surface area was recorded as 451.3 Å and 828.6 Å. The cavity volume exhibited by Ras protein was 593.8 Å and the surface area was 951.7 Å. The MEKK3 has a pocket volume of 484.8 Å and a surface area of 332.6 Å. The NFκB and the HRas-P21 had small pocket volumes of 327.4 Å and 224.3 Å, respectively, while the surface area was 283.1 Å and 336.6 Å. This method of identifying the specific properties of the pocket shape and size of the targets help to classify selective dietary scaffolds that aid in the design of anticancer drugs and the elaboration of molecular mechanisms.

### Dietary agents

3D and 2D structures of dietary agents such as emodin, eugenol, gingerol, sulforaphane, linalool, catechin, oleanolic acid, ursolic acid, curcumin, yakuchinone-A, pinusolide, α-boswellic acid, oleandrin, sesquiterpene lactone-326, resveratrol, triterpenoid, β-boswellic acid, anethole, capsaicin, glycolic acid, quercetin, genistein, ellagic acid, flavopiridol, zerumbone, garcinol, guggulsterone, parthenolide, halogenated monoterpenes, and silibinin have been successfully retrieved from PubChem with molecular formula, molecular weights and IUPAC structure identifiers (InChI and standard InChIKey) (Fig. S3). This was followed by the docking study on drug target to determine how these active principles form a molecular network by interfering with a specific target, which promotes the molecular mechanism for further optimization of novel anticancer compounds.

### Interactions of dietary agents with drug targets

**Figure 1:**
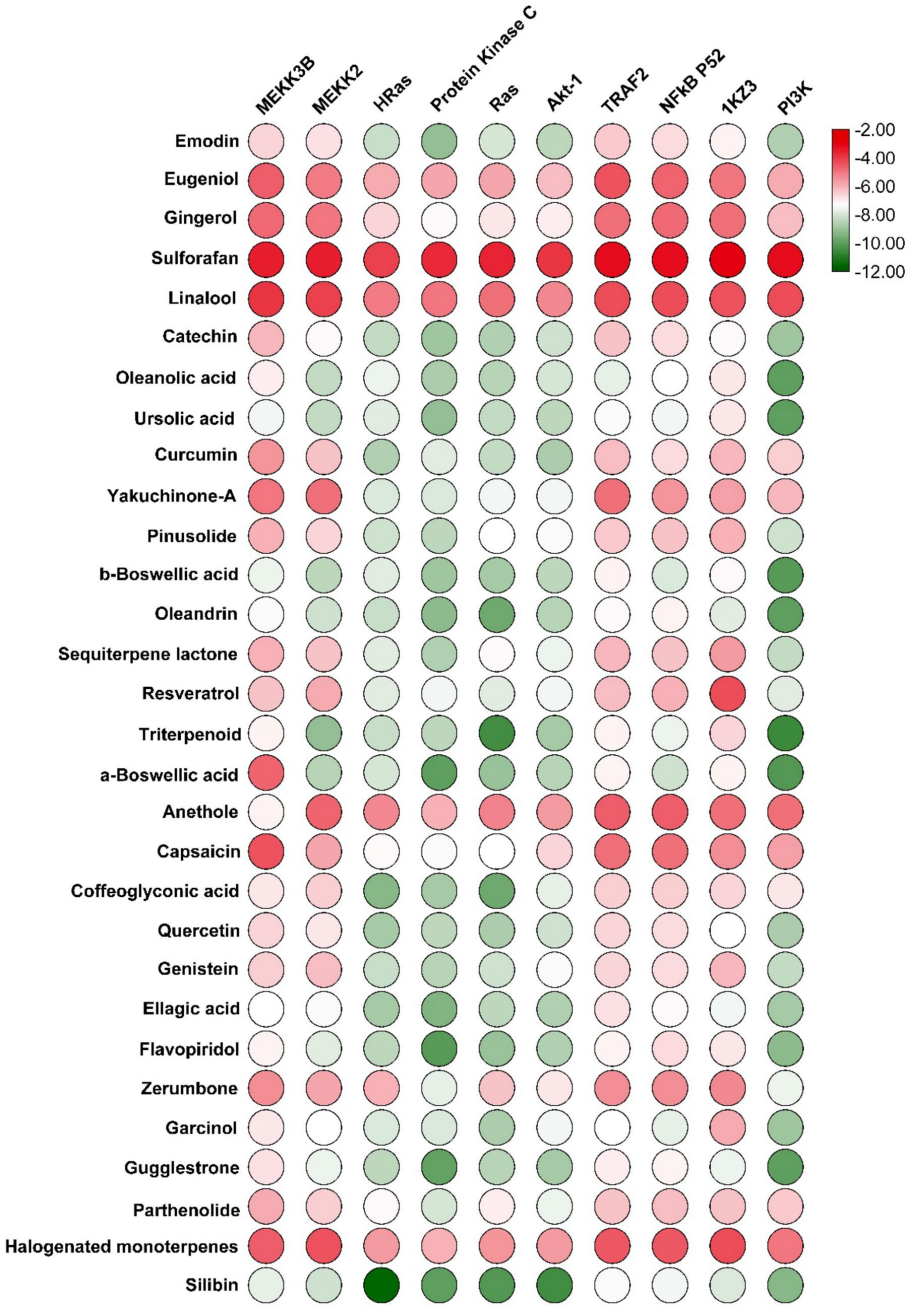
The heatmap represents the binding energies (kcal/mol) of the dietary compounds docked with various cancer drug-targets.

Determining the molecular partners of the dietary agent’s in the cellular context is a necessary approach to evaluate molecular mechanisms for effective treatment of cancer. To achieve this, therapeutic targets such as PKC-η, HRas-P21, AKT-1, Ras, PI3K-α, MEKK3, NFκB-P52, MEKK2b, and TRAF2 were subjected to docking studies to determine its molecular partner. Initially, the co-crystal ligands of the targets were extracted and the docking method was used to optimize the biologically relevant ligand binding conformation. For instance, the ligand PIK-108 is a propeller-shaped inhibitor and binds to the ATP binding pocket in the kinase domain of PI3K-α. However, the bound crystal ligand PIK-108 was extracted and docked into the pocket which produced nine biologically relevant docked poses of the ligand and was found to be identical by overlaying with the crystal ligand. Subsequently, to verifies the docking method, the RMSD of the docked pose was calculated to be 0.8 Å, indicating that the docking is reliable, consistent, and reproducible. Additionally, the ligand molecular network unveiled the phenyl moiety was oriented in the specificity site (Met_772_ and Trp_780_) and gated by the Met_772_ in the ATP pocket (Fig. S4). In the case of the dietary agents, the best binding energy have been found to be within − 9.0 to − 12.0 kcal mol^−1^, as well as molecular contacts were identified such as H-bonds, hydrophobic forces, electrostatic interactions, and Pi–Pi interactions with pocket residues (Fig. 1 and Table S3). Silibinin (− 9.3), flavopiridol (− 9.2), oleandrin (− 10.3), ursolic acid (− 10.0), α-boswellic acid (− 10.2), β-boswellic acid (− 10.1), triterpenoid (− 10.6), guggulsterone (− 10.0), and oleanolic acid (− 10.0) showed the highest binding affinity with PI3K-α. In the case of the PKC-η, silibinin (− 10.0), flavopiridol (− 10.1), oleandrin (− 9.2), ursolic acid (− 9.1), emodin (− 9.1), and ellagic acid (− 9.4) showed best binding energies. In addition, silibinin (− 10.2), flavopiridol (− 9.0), oleandrin (− 9.7), and α-boswellic acid (− 9.0) with the Ras, and silibinin (− 11.6) and glycolic acid (− 9. 3) compounds expressed substantial binding energies with the H-Ras, respectively. However, dietary compounds have provided the highest binding energy, but uncertainty with ligands has been observed due to unreliable binding poses, as well as interactions with pocket hotspot residues (Fig. S5). Finally, a potent polyphenolic flavonoid silibinin was identified as broad-spectrum inhibitory agent because it exhibited the highest binding energy and showed key interactions with H-Ras, PKC-η, Ras, and PI3K-α. The molecular interactions and superimposition studies suggest that silibinin binds to the ATP binding pocket of H-Ras, notably, it is shown to be identical to ATP. The silibinin-H-Ras complex revealed that the hydroxy-methoxy phenyl was placed in the hinge region, the benzodioxane resided at the ribose site, and the phosphate site was occupied by the dihydrochromen (Fig. S6). In the case of silibinin- PKC-η complex, it showed six bonds: the 3, 7 hydroxyl, and 1O groups of the benzopyran bonded with Asp_497_, Val_436_, Leu_486_, and the methoxyphenyl bonded with Asp_440_, whereas in the case of Ras-silibinin complex, it has been shown to have bonds of 7-OH and 1O atoms of the benzopyran with Lys_317_, Phe_228_, and Gly_213_, and the 1, 4 dibenzodioxins and an OH groups with Thr_235_. In addition, previous studies have reported a wide range of silibinin pharmacological activity, such as the STAT3 inhibitor^66^, anti-inflammatory, anti-PI3K-α and MAPK, cell cycle arrest at G_0_/G_1_ phase and p38 MAPK inhibitor^67, 68^, respectively. The silibinin have shown a broad-spectrum pharmacological effect on multiple drug targets and, therefore, can be utilized as a potential lead scaffold for further design of novel anticancer drugs with combined drug discovery and clinical study approach.

**Figure 2:**
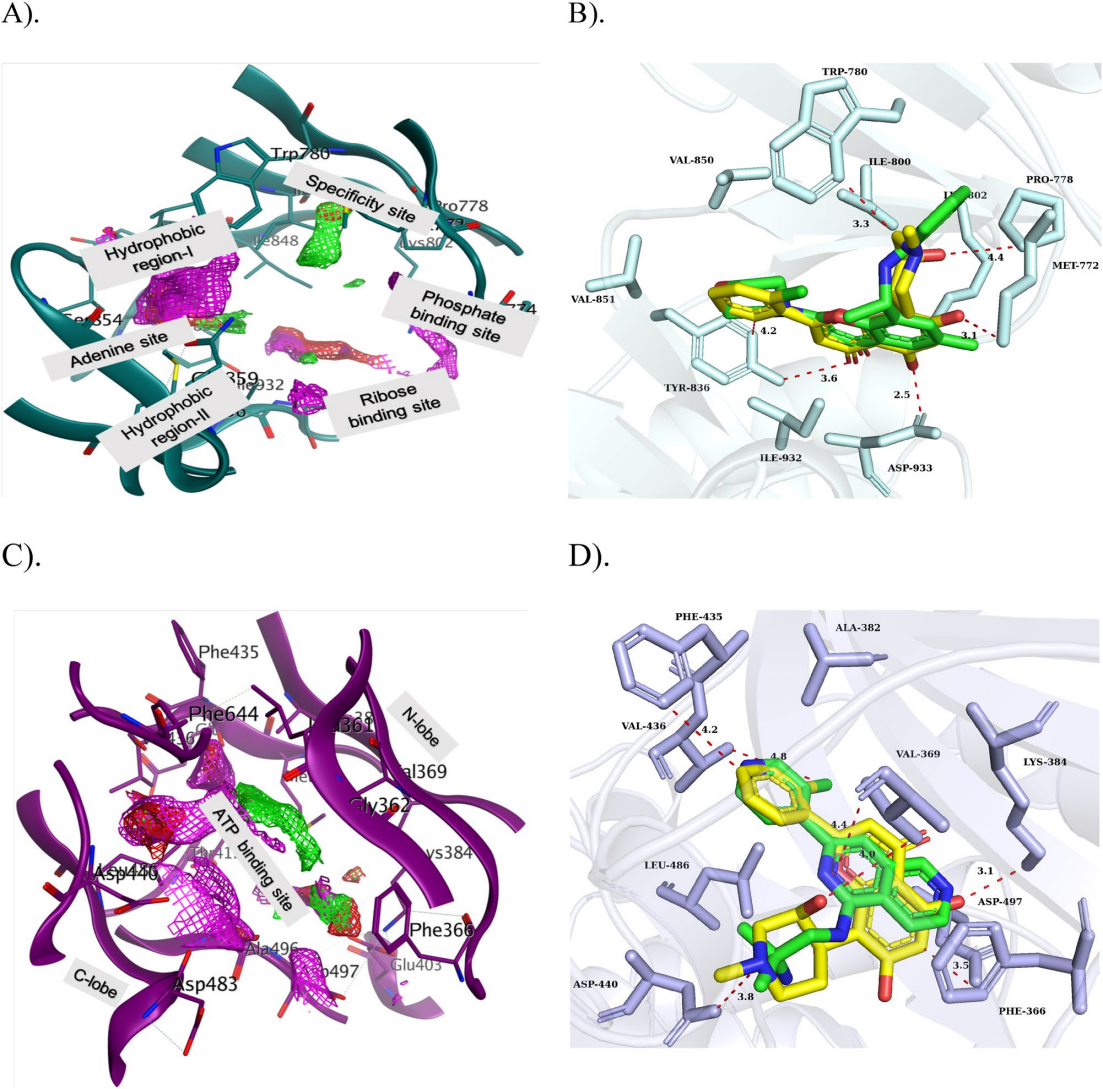
**(A)** 3D position of the sub-pocket in the ATP binding site of PI3K-α and **(B)** Overlays of bound crystal ligand PIK-108 (green) and flavopiridol (yellow) at the ATP binding site of PI3K-α. **(C)**. PKC-η is rendering ATP binding site with interaction potential including residues and **(D)** Overlays of bound crystal ligand naphthyridine (green) and flavopiridol (yellow) at the ATP binding site of the PKC-η. The proteins are shown in the cartoon and the key residues in the binding cavity indicate the sticks with labeling. Binding interactions are indicated in red dotted lines with distances (Å). (red: ligand exposure, green: hydrophobic, blue: hydrophilic).

Flavopiridol is a semisynthetic flavoalkaloid derived from the chrome alkaloid Rohitukie. Strikingly, flavopiridol has exerted significant two identical eloquent postures, as did the crystal ligand in the pockets of PI3K-α and PKC-η (Fig. 2). The PI3K-α isoform consists of two functional domains, the N-terminal and the C-terminal, and the ATP binding site is in the N-terminal domain with several sub-pockets: the adenine binding site (Ile_800_, Tyr_836_, Phe_930_, Met_922_), the hydrophobic site or the affinity pocket (Tyr_836_, Ile_848_, Ile_932_, Asp_810_) and the specificity site (Met_772_ and Trp_780_) is distinct in PI3K isoforms (Fig. 2A). However, flavopiridol formed critical interactions with ATP pocket of PI3K-α: two non-polar interactions were observed at the adenine binding site by the Tyr_836_ and the chlorobenzene, and at the specificity site by the Trp_780_ and the piperidine moiety. The 5-OH and 7-OH on the ring A formed polar interaction with Met_772_ and Asp_933_, as well as an O atom on the ring C and 3OH of piperidine with OH group of Tyr_836_ and Ile_932_ (Fig. 2B). Likewise, the molecular network of the PKC-η-flavopiridol complex has been described the chlorobenzene to be positioned at the adenine site by forming a non-polar contact with Phe_435_ and a halogen bond with the backbone of Val_436_. The A and C rings were inserted deep into the cavity and were able to form critical interactions with catalytic residues i.e., Lys_384_, Asp_497_ and Val_369_, and one Pi-Pi bond with Phe_366_. The piperidine formed an amide bond with Asp_440_ (Fig. 2C,D). Furthermore, the pharmacophore occupancy in the pockets of PI3K-α and PKC-η was described in the following section.

Furthermore, the Ras-flavopiridol complex revealed a non-polar contact of chlorobenzene with the Phe_228_, a polar bond was formed by the Asp_319_, the O-p bond was formed by 7-OH on the ring A and Tyr_232_, respectively. Polar and hydrophobic contacts between catalytic residues (Lys_317_ and Ala_218_) and piperidine (3OH) have been identified. Hitherto, previous studies on flavopiridol had reported several therapeutic activities, such as antitumor activity, by inhibiting the signal transduction pathway, as well as cyclin-dependent kinases (CDKs) as a strong binder, and induces apoptosis and anti-angiogenic activity in leukemic cells, respectively^69, 70^. Particularly, flavopiridol has been approved as an orphan drug for chronic myeloid leukemia^71^. Given these broad-spectrum pharmacological profiles of dietary agents as evidenced by acting on multiple targets, in this case the isoform-selective inhibitors for PI3K-α and PKC-η were designed based on different scaffolds.

### Complex based pharmacophore modeling

**Figure 3:**
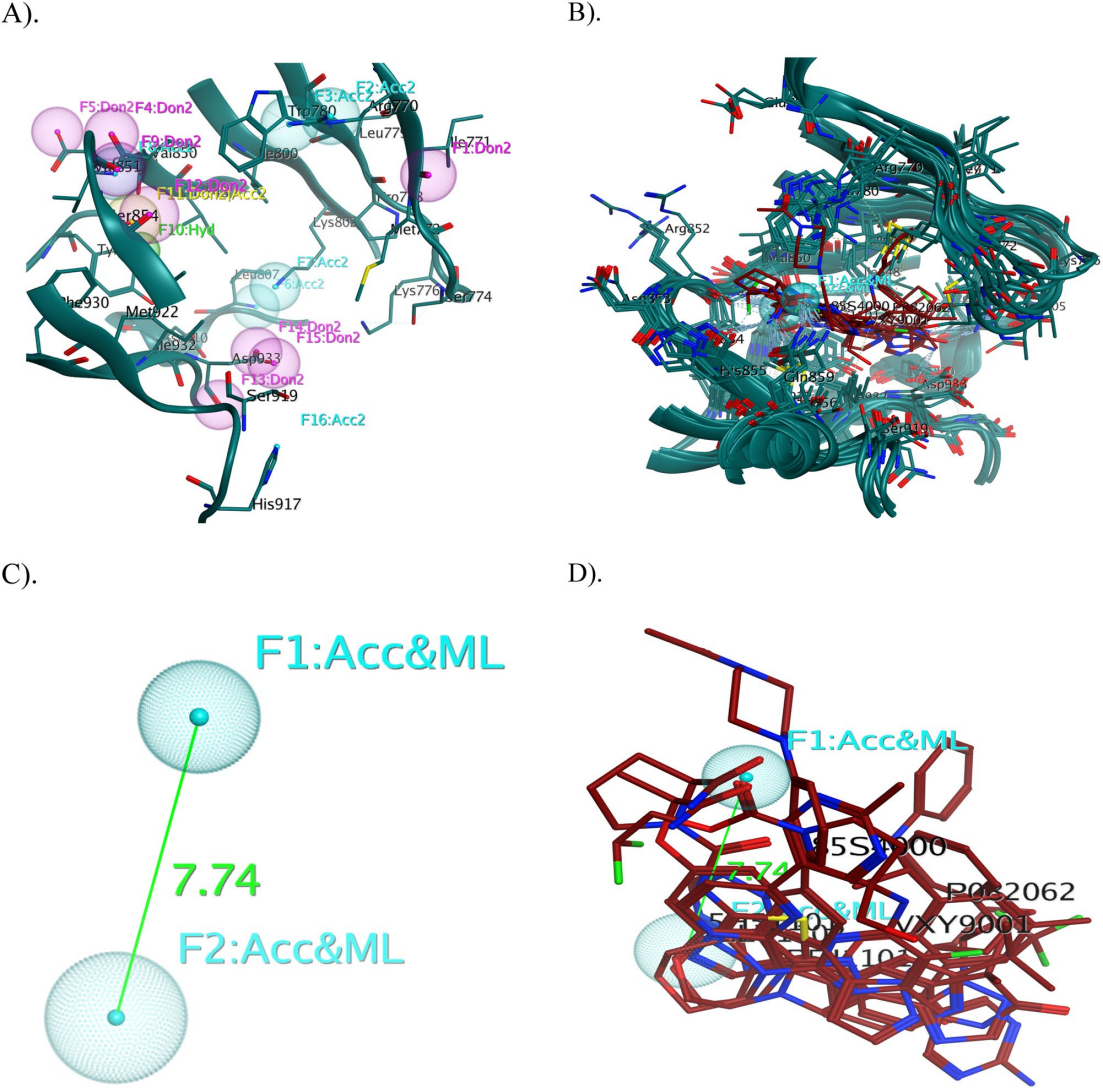
^.^ **(A)** 3D pharmacophore annotation of ATP binding pocket of PI3K-α, **(B)** Overlay of crystal structures of PI3K-α co-crystalized with different ligands and generated 3D spatial arrangement of pharmacophore features with vector projections in the binding cavity of PI3K-α, **(C)** The distance constraints were calculated between the vital features, and **(D)** The co-crystalized ligands of PI3K-α were superimposed and aligned with the generated pharmacophore query. The proteins are shown in the cartoon and the key residues in the binding cavity indicate the sticks with labeling and the ligands are indicated in red colour. (Don & Acc: hydrogen bond donor/acceptor, Aro: aromatic center, Don: hydrogen bond donor, Acc: hydrogen bond acceptor, Hyd: hydrophobic, Cat & Don: cation donor).

The pharmacophore modeling is a pioneering approach to drug discovery campaign, based primarily on physical–chemical features, shape, volume, and 3D alignment of the pocket. Pharmacophore is a combined steric and electronic descriptor of the drug, which has a specific inhibitory action on therapeutic targets. First and foremost, for a clear understanding of the pharmacophoric properties of the active site, a pharmacophore map for the ATP binding pocket of PI3K-α was constructed, which revealed sixteen pharmacophores and represented in Fig. 3A. In addition, crystal structures of PI3K-α co-crystalized with different ligand were downloaded from PDB (Table 1). The PLIF method summarized the crucial interactions from the complexes using a fingerprint scheme, which reveals binding site residues interact with which parts of the ligands. In this scenario, the PLIF detects eleven key residues in the pocket, namely Met_772_, Ser_774_, Lys_802_, Asp_810_, Tyr_836_, Glu_849_, Val_851_, Ser_854_, Gln_859_, Met_922_ and Ile_932_, respectively, interacting with different ligands (Fig. S7)^72–74^. The PLIF enable pharmacophore queries based on the frequencies of protein–ligand contacts; therefore, two pharmacophore features such as F1 (Acc & ML) and F2 (Acc & ML) were generated by the PILF and these two features have been converted to the searchable pharmacophore query, respectively (Fig. 3B). The geometric distance and angle constraints of key pharmacophoric features on the 3D pharmacophoric map were calculated and overlaid with superposition of different ligands (Fig. 3C,D).

**Table 1:** Crystal structures of PI3K-α and PKC-η co-crystalized with different ligands were downloaded from PDB.

S. No	Crystal structures (PDB ID)	Ligand	Resolution	Year
**PI3K-α**				
**1**	4A55	PIK-108	3.5 Å	2011
**2**	4JPS	(2S)-N ~ 1 ~ -{4-methyl-5-[2-(1,1,1-trifluoro-2-methylpropan-2-yl)pyridin-4-yl]-1,3-thiazol-2-yl}pyrrolidine-1,2-dicarboxamide	2.20 Å	2014
**3**	5DXT	(2S)-2-({2-[1-(propan-2-yl)-1H-1,2,4-triazol-5-yl]-5,6-dihydroimidazo[1,2-d][1,4]benzoxazepin-9-yl}oxy)propanamide	2.25 Å	2016
**4**	5UBR	1-[4-(3-{4-amino-5-[1-(oxan-4-yl)-1H-pyrazol-5-yl]pyrrolo[2,1-f][1,2,4]triazin-7-yl}phenyl)piperazin-1-yl]ethan-1-one	2.40 Å	2017
**5**	6PYS	(3S)-3-benzyl-3-methyl-5-[5-(2-methylpyrimidin-5-yl)pyrazolo[1,5-a]pyrimidin-3-yl]-1,3-dihydro-2H-indol-2-one	2.19 Å	2019
**6**	7K6M	2,2-difluoroethyl (3S)-3-{[2'-amino-5-fluoro-2-(morpholin-4-yl)[4,5'-bipyrimidin]-6-yl]amino}-3-(hydroxymethyl)pyrrolidine-1-carboxylate	2.41 Å	2021
**PKC-η**				
**1**	3TXO	2-methyl-N ~ 1 ~ -[3-(pyridin-4-yl)-2,6-naphthyridin-1-;**[**yl]propane-1,2-diamine	2.05 Å	2011
**2**	3TXO	Staurosporine	2.05 Å	2011

**Figure 4:**
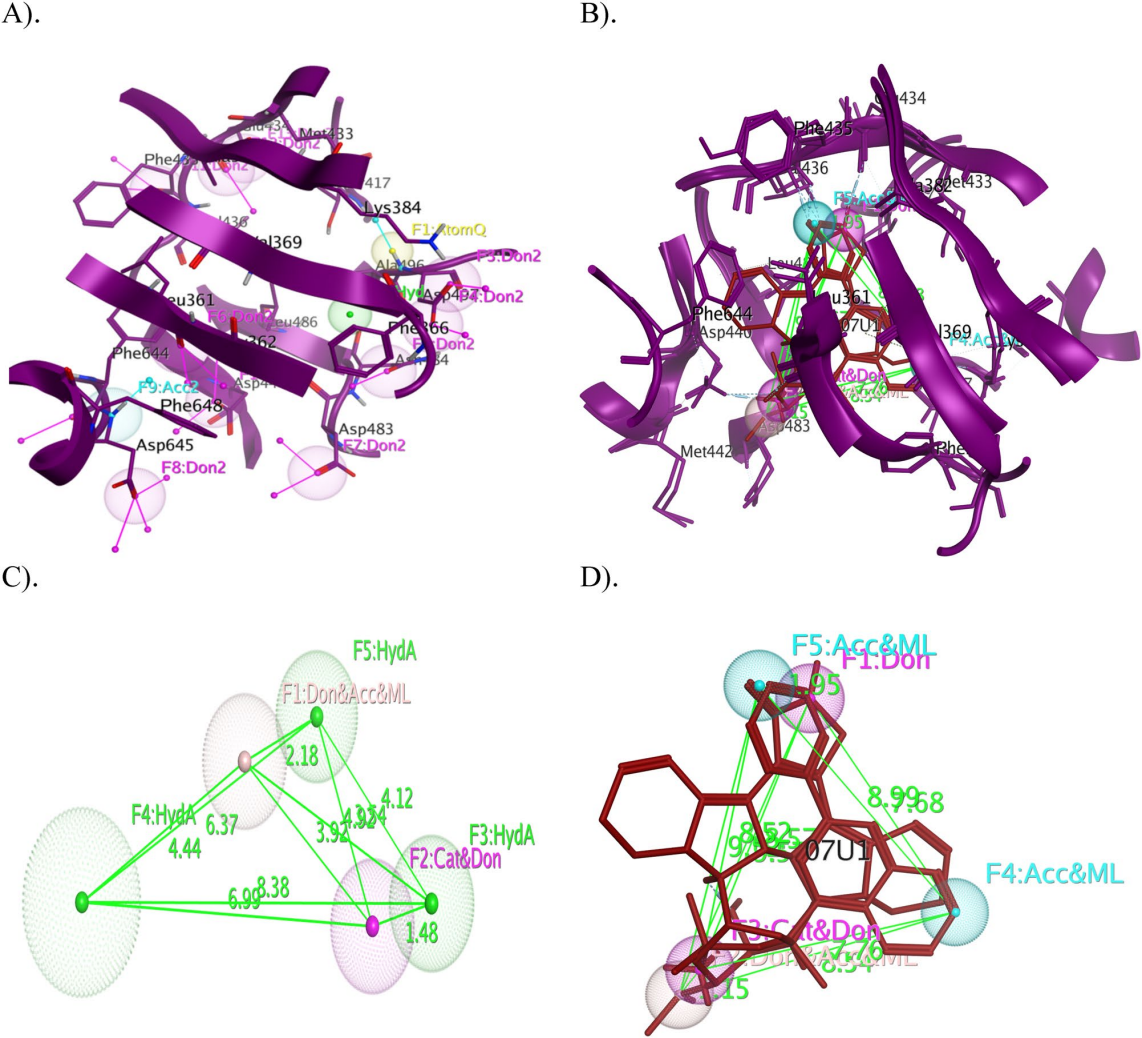
**(A)** 3D pharmacophore annotation of ATP binding pocket of PKC-η, **(B)** Overlay of crystal structures of PI3K-η co-crystalized with different ligands and generated 3D spatial arrangement of pharmacophore features with vector projections in the binding cavity of PI3Kη, **(C)** The distance constraints were calculated between the vital features, and **(D)** The cocrystallized ligands of PKC-η were superimposed and aligned with the generated pharmacophore query. The proteins are shown in the cartoon and the key residues in the binding cavity indicate the sticks with labeling and the ligands are indicated in red colour. (Don & Acc: hydrogen bond donor/acceptor, Aro: aromatic center, Don: hydrogen bond donor, Acc: hydrogen bond acceptor, Hyd; hydrophobic, Cat & Don: cation donor).

Similarly, a pharmacophore map for the PKC-η was constructed, which exhibited thirteen pharmacophore features within the ATP binding pocket (Fig. 4A). However, to create the PLIF, two crystal structures were used, one complexed with 07U and the other docked complex with staurosporine. A PLIF reveals crucial contacts with hotspot residues, namely Leu_361_, Asp_440_, Asp_483_, Leu_486_, Ala_496_ and Asp_497_, and were represented by as a barcode and population in graphical mode (Fig. S8)^75^. Subsequently, the essential pharmacophore query has been resolved by using the PILF of PKC-η (Fig. 4B). Unambiguously, five key pharmacophoric features—Don (F_1_), Cat & Don (F_2_), Hyd (F_3_), Hyd (F_4_) and Hyd (F_5_) have been identified to be interacted with hotspot residues and identified as a virtual filter to screen chemical library; in addition, the distance and angle limits of geometrically identified pharmacophoric features were measured on a 3D pharmacophoric map (Fig. 4C, D).

### Database screening

**Figure 5:**
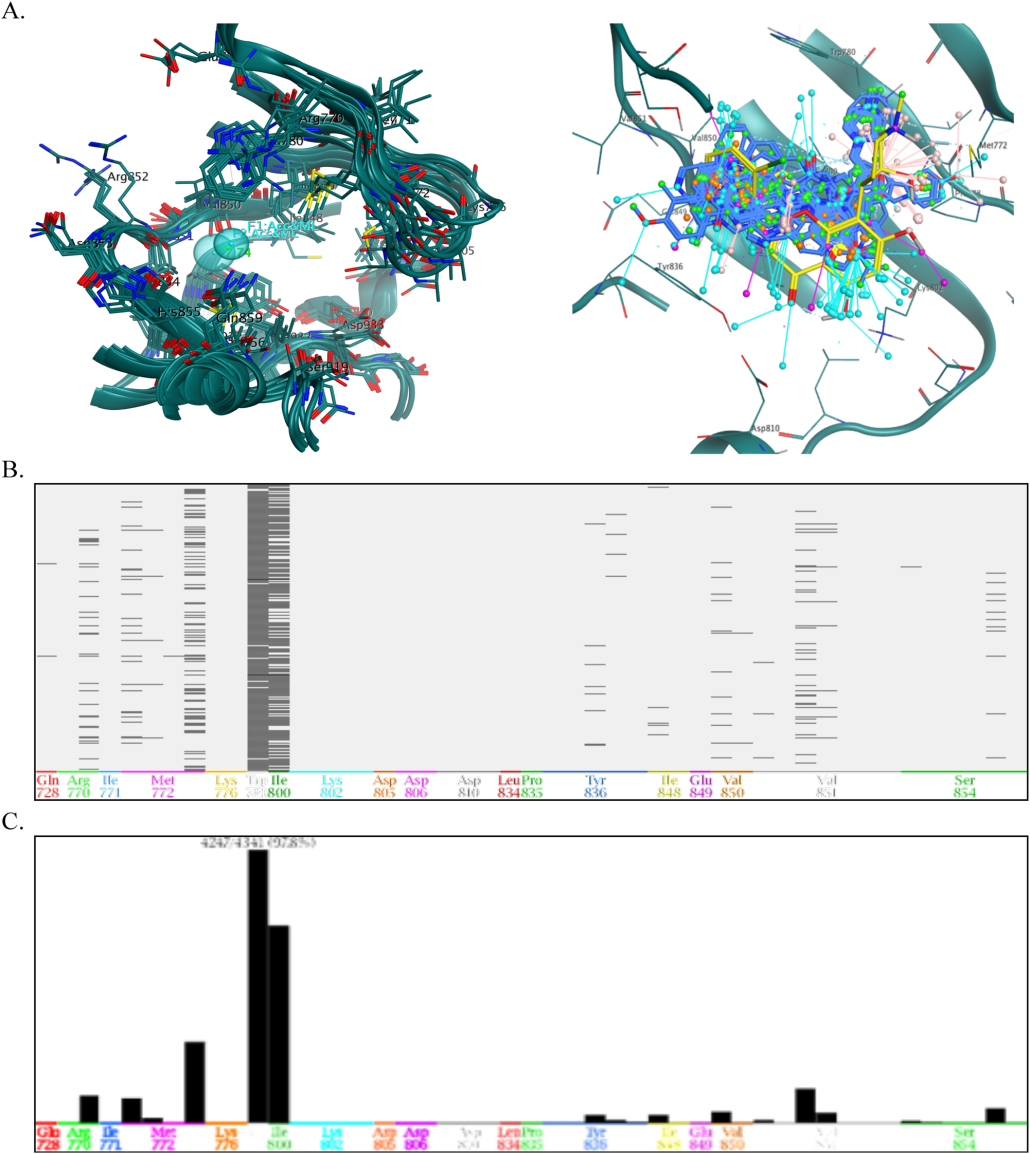
^.^ **(A)** The distance constraints of vital 3D pharmacophoric features were calculated and displayed in the ATP binding site of PI3K-α and Overlays of virtual hits in the ATP binding site of PI3K-α including vector projections. The PLIF computed the interactions between the PI3Kα-virtual, **(B)** the barcode representation of fingerprint of the PI3K-α-ligand complexes: The x-axis displays a three-letter code of the key residues and the y-axis shows the number of PI3K-α ligand complexes, and **(C)** population mode refers to the histogram of fingerprint of the virtual hits showing the number of ligands with which each residue interacts.

**Figure 6:**
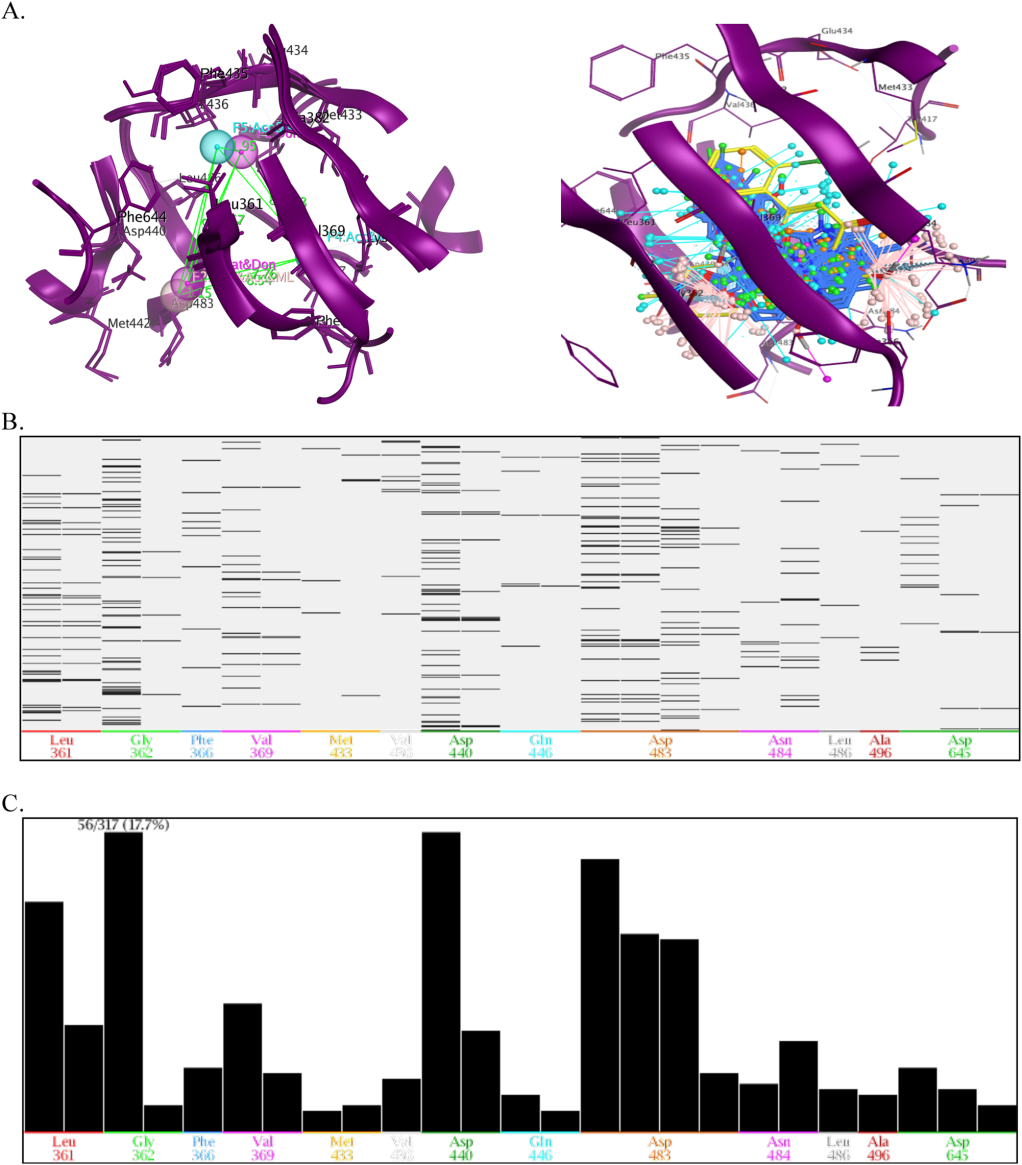
^.^ **(A)** The distance constraints of vital 3D pharmacophoric features were calculated and displayed in the ATP binding site of PKC-η and Overlays of virtual hits in the ATP binding site of PKC-η including vector projections. The PLIF computed the interactions between the PKC-η PKC-η -virtual, **(B)** The barcode representation of fingerprint of the PKC-η-ligand complexes: The x-axis displays a three-letter code of the key residues and the y-axis shows the number of PKC-η-ligand complexes, and **(C)** Population mode refers to the histogram of fingerprint of the virtual hits showing the number of ligands with which each residue interacts.

Virtual screening is a versatile approach to screen large chemical libraries by applying various filters, for instance, the pharmacophoric strategy of retrieving potential leads with specified therapeutic properties. The validated 3D pharmacophore query of PI3K-α and PKC-η was utilized to screen the refined ChemBridge database. Due to virtual screening 6048 pharmacophoric hits molecules were obtained for the PI3K-α and 4069 for the PKC-η and all possible pharmacophoric hits with that query pharmacophores by fitting over the active pocket (Figs. 5A, 6A). In addition, the PLIF scheme summarizes the highest percentage of interaction between the ligands and the protein targets with 97% of virtual hits showing key contacts with hotspot residues of PI3K-α (Fig. 5B,C), whereas 17% hits were shown for PKC-η (Fig. 6B,C). However, to classify these perspective lead compounds from the pharmacophoric hits, which were further subjected to various constraints like docking, MM-GBVI, Lipinski rule of five, ADMET and toxicity properties. Henceforth, the top-rank ligands were sorted out and provided complex interactions with key residues, unambiguously, the ten best promising lead compounds for PI3K-α, exhibiting binding energy from − 10.0 kcal mol^−1^ to − 8.5 kcal mol^−1^, thirteen compounds for PKC-η showed binding energy from − 11.5 kcal mol^−1^ to − 8.9 kcal mol^−1^, respectively (Table S4 and S5).

### Born interaction energies and binding affinities

**Figure 7:**
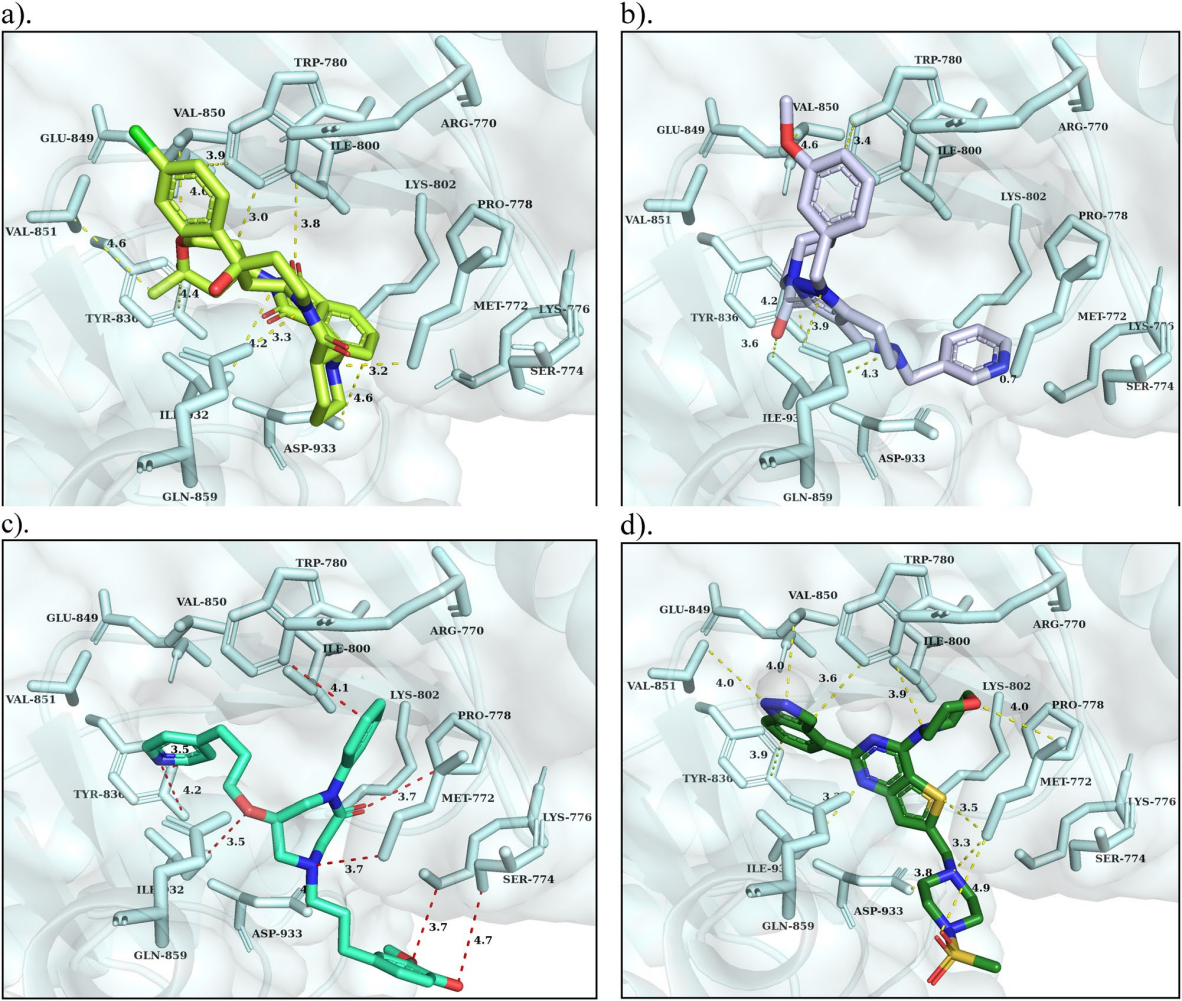
^.^ Binding mode, interactions of **(a)** Compound-1, **(b)** Compound-2, **(c)** Compound-3 and **(d)** Pictilisib (GDC-0941 (reference inhibitor)) at the ATP binding pocket of PI3K-α. The protein (cyan) is shown in the cartoon with the surface model, the active site residues are indicated in sticks with labeling and the binding interactions are indicated in red dotted lines with distances (Å).

The cohesive relationships of born interaction forces and binding affinities of the top-rank scaffolds were calculated using the generalized born/volume integral method available in MOE. Precisely, the binding affinity (kcal mol^−1^) was calculated after minimization the energy of the complexes, resulting in three lead scaffolds for PI3K-α, seven selected for PKC-η (Tables 2 and 3). Henceforth, the molecular network of complexes has been applied to detect implicated residues by tether restraint by 2D assessment, which excludes false positive due to presumption and default in docking procedures along with scoring methods. Furthermore, significant 3D spatial alignment and significant molecular interactions of lead scaffolds were achieved with the pocket of PI3K-α and PKC-η. In the case of the PI3K-α, compound-1 showed the highest binding energy of − 10.5 kcal mol^−1^ by exhibiting vital contacts such as the 2, 3 dihydro-1H isoindole1 and the 3dione functional groups bound to an atom O of Val_850_ and Ile_800_, an N-atom forms a bond with Trp_780_, a hydrophobic contact with Tyr_836_ and one bond with Val_851_, respectively. Chlorobenzene displayed a hydrophobic contact with Trp_780_. The carbonyl piperidine gave a hydrophobic interaction and showed a polar bond with the carbonyl of Lys_802_, an N-atom bound to Ile_932_, and the 4-OH interaction of piperidine formed a bond with Asp_933_ and Met_772_ (Fig. 7a). Compound-2 exhibits binding energy of − 8.5 kcal mol^−1^ and forms seven bonds: two hydrophobic interactions with Trp_780_ and Tyr_836_, the isoquinoline and the benzodioxol functional groups exhibit four bonds with Tyr_836_, Val_851_, Ile_932_ and Met_772_, the hydroxy-piperidine showing association with S-atom of Met_772_ (Fig. 7b). The compound-3 displayed the binding energy of − 8.7 kcal mol^−1^ and formed nine binding interactions. The pyridine exhibited two bonds such as a polar and a hydrophobic contact with OH of Tyr_836_. Oxygen atom bonds with Ile_832_ and, 1, 4‐diazepan‐2‐one group formed three bonds with Asp_933_, Met_772_ and Pro_778_. The phenyl group hydrophobically bound to Trp_780_. The 4-hydroxy3-methoxy phenyl formed two bonds with Ser_774_ and Lys_776_ (Fig. 7c). In addition, pictilisib is a potent PI3K inhibitor, displaying the binding energy of − 8.5 kcal mol^−1^ and exhibiting eleven binding interactions with the ATP binding site residues of PI3K-α (Fig. 7d). The primidine formed four bonds: two hydrophobic interactions with Tyr_836_ and Trp_780_ and two polar bonds with Val_850_ and Val_851_. The methyl sulfonyl group binds to Asp_933_ and Met_772_ and the 4-morpholinyl formed two binds to both Met_772_ and Ile_800_. The piperazinyl moiety exhibited two bonds with Ile_932_ and Met_772_.

**Table 2:** 2D structures, IUPAC name, binding interactions, efficiency, binding energy, binding affinity, and MM/GBVI of the best lead molecules of PI3K-α.

S. No	IUPAC Name	Interactions	Efficiency	Binding energy (kcal/mol)	Binding affinity (pKi)	MM/GBVI (kcal/mol)
1	4-{3-[4-(4-chlorophenyl)-4-hydroxypiperidine-1-carbonyl]piperidin-1-yl}-2-(2,2-dimethyloxan-4-yl)-2,3-dihydro-1H-isoindole-1,3-dione	Tyr836----arene	4.0	− 10.5	7.7	− 22.8
		Val850----OH	4.6			
		Trp780----NC	3.0			
		Ile932----NC	4.2			
		Ile800----OH	3.8			
		Lys802----OH	3.2			
		Met772----NC	3.2			
		Asp933----OH	4.6			
		Val851----C	4.6			
		Tyr836----C	4.4			
2	1‐{1‐[(3‐methoxyphenyl)methyl]‐5‐[(pyridin‐3‐ yl)methyl]pyrazolo[4,3‐c]pyridine‐3‐ carbonyl}pyridin‐3‐o	Tyr836----arene	4.2	− 8.5	7.2	− 20.9
		Tyr836OH----arene	2.8			
		Trp780----arene	3.4			
		Ile932----N	3.6			
		Met772----O	0.7			
		Gln859----N	3.9			
3	4‐[3‐(4‐hydroxy‐3‐methoxyphenyl)propyl]‐1‐phenyl‐6‐ [(pyridin‐4‐yl)methoxy]‐1,4‐diazepan‐2‐one	Trp780----arene	4.1	− 8.7	7.2	− 23.6
		Pro778----O	3.7			
		Tyr836----arene	3.4			
		Tyr836OH----N	3.5			
		Ile832----O	3.5			
		Asp933----N	4.0			
		Met772----N	3.7			
		Lys776----O	3.7			
		Ser774----O	4.7			
4	Pictilisib	Tyr836----arene	3.6	− 8.5	7.0	− 22.4
		Val851----N	4.0			
		Val850----N	4.0			
		Trp780----arene	3.6			
		Ile800----N	3.9			
		Met772----S	3.5			
		Met772----N	3.3			
		Met772----O	4.9			
		Met772----O	4.0			
		Asp933----N	3.8

**Table 3:** 2D structures, IUPAC name, binding interactions, efficiency, binding energy, binding affinity, and MM/GBVI of the best lead molecules of PKC-η.

Compound	IUPAC name	Interactions	Efficiency	Binding energy (kcal/mol)	Binding affinity (pKi)	MM/GBVI (kcal/mol)
1	7‐(1,3‐benzothiazol‐2‐yl)‐4‐[1 (2hydroxyphenyl)ethenyl]‐2,3,4,5‐tetrahydro‐1,4‐benzoxazepin‐9‐ol	Phe435----arene	3.6	−10	7.7	−31.8
		Leu486----S	3.5			
		Val369----N	4.7			
		Asp497----O	3.2			
		Phe366----arene	4.3			
2	4(1aminocyclobutanecarbonyl)‐7‐(1,3‐benzothiazol‐2‐yl)‐2,3,4,5‐tetrahydro‐1,4‐benzoxazepin‐9‐ol	Phe435----arene	3.8	−9.9	10.0	−21.0
		Leu486----S	3.9			
		Val369----N	3.6			
		Asp497----O	3.2			
		Phe366----arene	4.1			
3	4‐{1‐[9‐hydroxy‐7‐(4‐methylphenyl)‐2,3,4,5‐ tetrahydro‐1,4‐benzoxazepin‐4‐yl]ethenyl}benzene‐ 1,3‐diol	Phe435----arene	4.6	−9.4	9.1	−24.7
		Val436----H	3.0			
		Val369----O	4.7			
		Asp497----O	3.9			
		Phe366----arene	4.4			
		Asp440----N	3.7			
		Asp440----O	2.8			
		Asp440----O	4.3			
4	7‐(3,6 dimethylpyrazin‐2‐yl)‐4‐(3‐hydroxybenzoyl)‐ 2,3,4,5‐tetrahydro‐1,4‐benzoxazepin‐9‐ol	Phe435----arene Val436----N Val436----N Leu486----H Val369----H Lys384----O Asp497----O Phe366----arene Asp440----O Asp497----O	4.2 3.5 3.9 4.0 4.1 4.6 3.3 3.8 3.8 4.4	− 9.4	7.9	−22.1
5	1‐{4H‐[1,2'‐bipyrazin]‐4‐yl}‐3‐(2‐hydroxyphenyl)‐3(4hydroxyphenyl)propan‐1‐one	Phe435----arene	3.9	−9.4	10.6	−27.2
		Ala382----O	4.7			
		Ile486----O	2.8			
		Lys384----O	3.3			
		Asp440----O	3.8			
		Asp440----N	3.9			
		Phe366----arene	3.6			
6	4‐[(4‐hydroxy‐5‐methoxypyridin‐2‐yl) methyl]‐7‐(4‐methylphenyl)‐2,3,4,5‐tetrahydro‐1,4‐benzoxazepin‐9‐ol	Phe435----arene	3.9	−9.3	8.0	−21.1
		Val436----H	2.8			
		Lys384----O	4.2			
		Val369----O	4.8			
		Phe366----arene	4.3			
		Asp440----O	3.5			
		Asp440----O	4.1			
		Asp440----N	4.5			
7	7‐(5‐chloropyridin‐2‐yl)‐4‐(4‐hydroxy‐1H‐pyrrole‐2‐carbonyl)‐2,3,4,5‐tetrahydro‐1,4‐benzoxazepin‐9‐ol	Phe435----arene	3.8	−9.3	8.6	−21.6
		Val436----O	2.3			
		Ala382----N	4.1			
		Leu486----N	4.7			
		Val369----O	4.7			
		Val369----N	4.2			
		Val369----N	4.3			
8	Staurosporine	Val436----O	2.6	−8.5	7.4	−18.7
		Leu486----N	3.8			
		Leu486----O	3.2			
		Val369----N	4.7			
		Val369----N	3.9			
		Phe366----arene	4.6			
		Asp440----N	2.7

**Figure 8:**
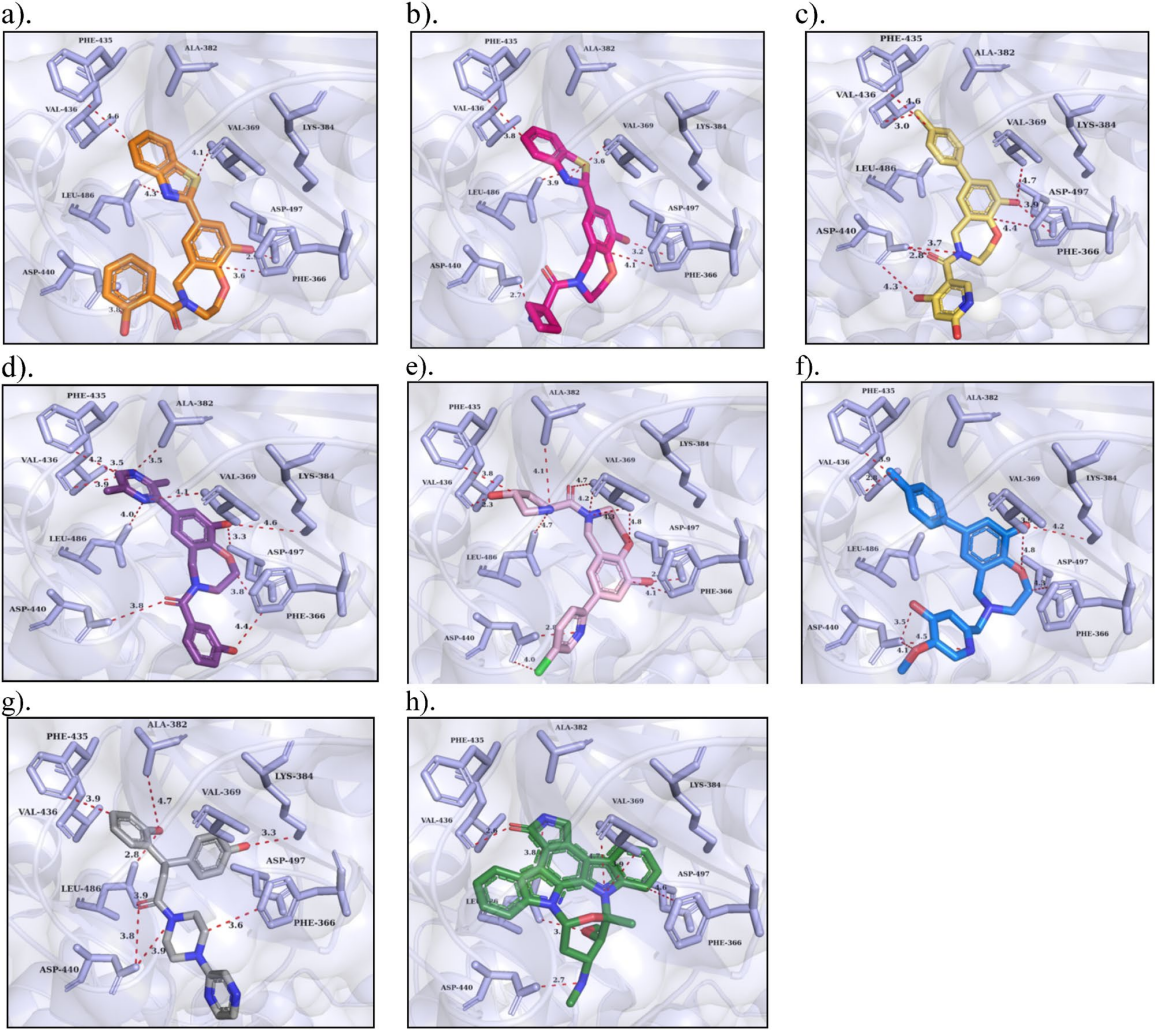
^.^ Binding mode, interactions of **(a)** Compound-1, **(b)** Compound-2, **(c)** Compound-3 and **(d)** Compound-4, **(e)** Compound-5, **(f)** Compound-6, **(g)** Compound-7, and **(h)** Staurosporine (reference inhibitor) at the ATP binding pocket of PKC-η. The protein (light blue) is shown in the cartoon with the surface model, the active site residues are indicated in sticks with labeling and the binding interactions are indicated in red dotted lines with distances (Å).

In contrast, an estimate of lead optimization revealed seven lead scaffolds for PKC-η and a molecular network with ATP binding residues. Compound-1 displayed the highest binding energy is − 10.0 kcal mol^−1^ and the 4-hydroxy phenyl group exhibited two bonds with Phe_435_ and Ala_382_, the carbonyl moiety bonded with Leu_486_, the benzoxazepine-9-ol group bound with Val_369_, Lys_384_, and Asp_497_, a hydrophobic bonding with Phe_366_, and the benzothiazole showing polar bonding with Asp_440_ have been observed (Fig. 8a). Similarly, compound-2 exhibited the highest binding energy of − 9.9 kcal mol^−1^ by exhibiting molecular contacts such as the hydrophobic bonding with Val_436_, Val_369_, and Leu_486_ have been examined by the 1, 3 benzothiazoles, whereas the benzoxazepine-9-ol was found to bind to Asp_497_ and Phe_366_, and the amino group of cyclobutene carbonyl was found to bind to Asp_440_, respectively (Fig. 8b). Compound-3, 4 and 5 showed the binding energy of − 9.4 kcal mol^−1^, while compounds 6 and 7 showed the binding energy of − 9.3 kcal mol^−1^. However, compound-3 shows eight interactions—the dimethyl pyrazine formed a Pi-Pi bond with Phe_435_ and a polar bond with the carboxylic group of Val_436_, whereas the benzoxazepine displaying four bonds such as a pi-pi bond with Phe_366_ as well as three polar bonds with Val_369_, Asp_497_ and Asp_440_, and the hydroxy-benzoyl exhibit two bonds with Asp_440_, respectively (Fig. 8c). Compound-4 showed the highest binding interactions—the dimethyl pyrazine group was positioned at the adenine site and formed six bonds such as a Pi-Pi bonding with Phe_435_ and five polar bonds with the backbones of Val_436_ and Val_369_ and so on with the side chains of Ala_382_ and Leu_486_. The benzoxazepine exhibited interactions with catalytic residues of Lys_384_ and Asp_497_, a Pi-Pi bond with Phe_366_, and the 3‐hydroxy benzoyl showed two bonds: a polar bond with Asp_440_ and a Pi-cationic bond with Phe_366_ (Fig. 8d). Compound-5 formed twelve bonds: the hydroxy-pyrrole was positioned at the adenine site and explicated bonds with the carboxylic group of Val_436_, a Pi–Pi bond with Phe_436_, and two bonds with the side chains of Ala_382_ and Leu_486_. The benzoxazepine showed five bonds such as four polar bonds with Val_369_ and Asp_497_, and a Pi-Pi bond with Phe_366_, and the chloro-pyridine displayed two bonds with Asp_440_ (Fig. 8e). Compound-6 showed eight bonds: the methyl phenyl group interacted with Val_436_ and showed a Pi-Pi bond with Phe_436_. The benzoxazepine formed four bonds: two bonds with catalytic residues of Lys_384_ and Asp_497_, and a Pi–Pi bond with Phe_366_. The hydroxy methoxy pyridine formed three bonds with Asp_440_ (Fig. 8f). Compound-7 disclosed seven interactions: the 2-hydroxyphenyl was positioned at the adenine pocket and formed two polar bonds with Leu_486_ and Ala_382_ and a Pi–Pi bond with Phe_436_. The 4-hydroxy phenyl propane formed complex interactions with catalytic residues of Lys_384_ and Asp_440_, while the bi-pyrazine formed a polar bond with Asp_440_ and a Pi-Pi bond with Phe_366_ (Fig. 8g). In addition, the reference drug staurosporine is a natural alkaloid and ATP antagonist, which deeply occupied at the ATP binding site by releasing the binding energy of − 8.5 kcal mol^−1^ and showed five polar and a non-polar interaction, respectively (Fig. 8h). An oxygen atom of lactam formed one polar interaction with Val_436_, Nitrogen atom of triazocyclo noncosa formed two bonds with Val_369_ and one non-polar contact with Phe_366_, methoxy of tetrahydropyran exhibited one bond with Leu_486_ and methylamine bonded with Asp_440_, respectively.

### Drug-likeness and toxicity properties

**Figure 9:**
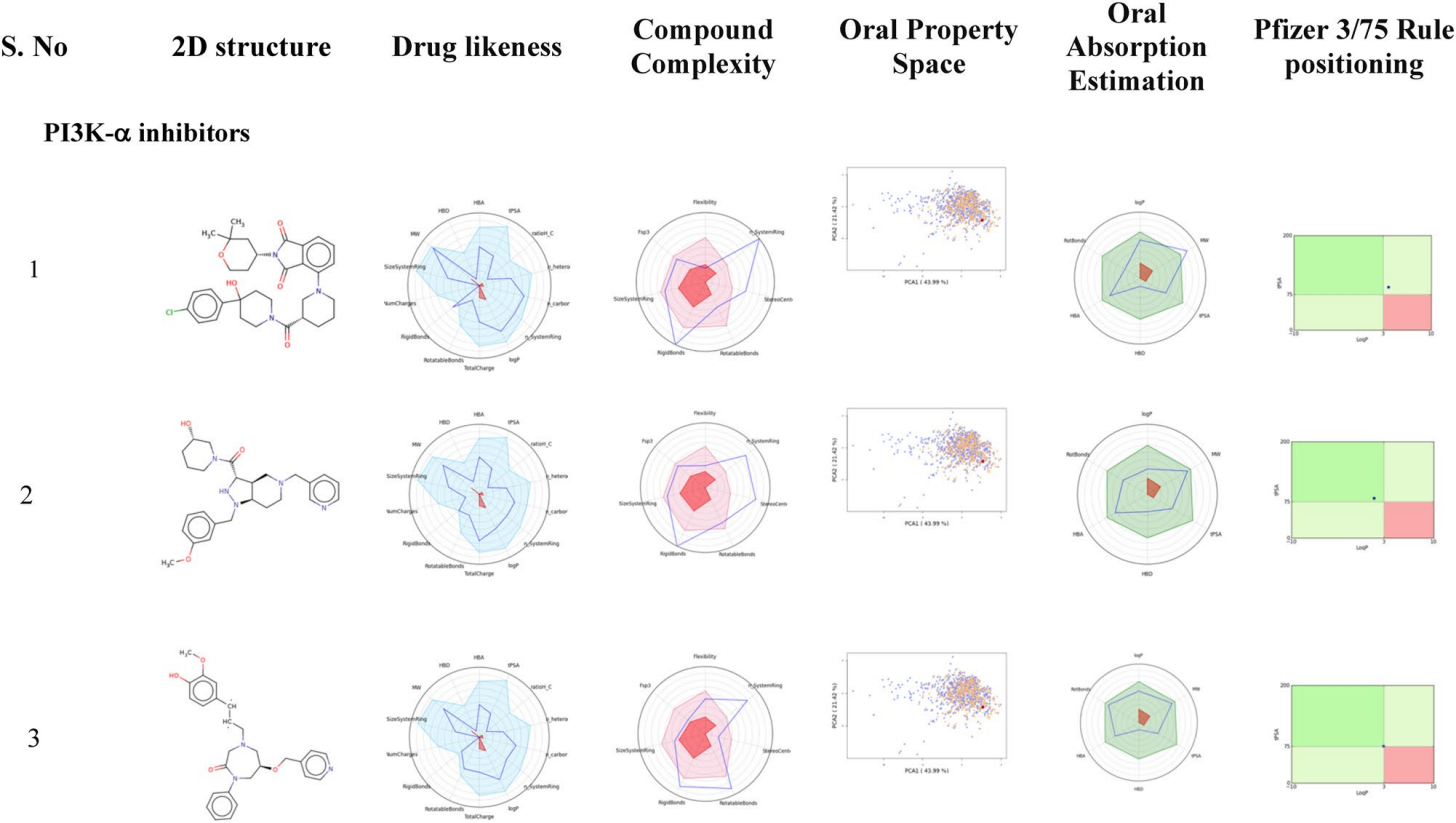

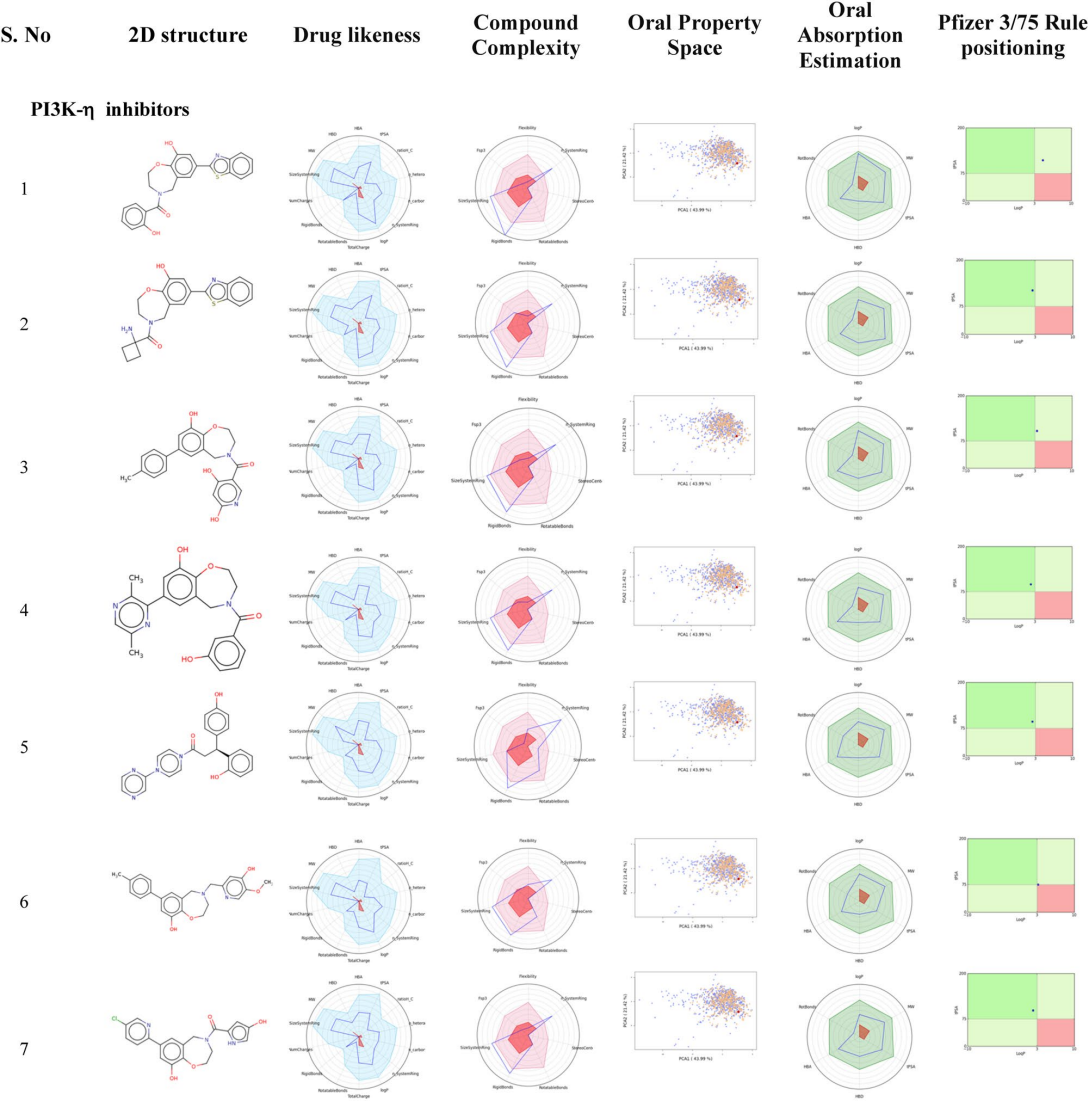
^.^ 2D structure, drug-likeness, compound complexity, oral property space, oral absorption estimation, and Pfizer 3/75 rule positioning of the lead molecules of PI3K-α and PKC-η were assessed by using FAF-Drugs and Swiss-ADME server.

The molecular properties of the drug, pharmacodynamics, pharmacokinetics, ADMET, and toxic properties play a crucial role in bioavailability. Therefore, the identified isoform selective lead scaffolds were evaluated using a FAF-Drugs server (Fig. 9; Table 4). Basically, the pharmacodynamics properties revealed promising molecular interactions with decisive residues of the pocket. Lipinski’s rule: molecular weight, cLogP, hydrogen bond acceptors and donors were measured in relation to the physico-chemical and the pharmacokinetic properties of the leads. The molecular weight was estimated to be less than 500 (≤ 500) and the cLogP or partition coefficient plays a major role in accessing the drug in the body and was predicted to be less than five (≤ 5) indicating appropriate absorption and distribution properties. In addition, H-bond acceptors were found to be less than ten (≤ 10), while H-bond donors were less than five (≤ 5), resulting in the lead compounds obeyed the rule of five. Furthermore, it has been revealed that absorption capacity was soluble and moderately soluble by estimating the solubility in water. Estimation of interactions with skin permeability, gastrointestinal absorption, blood–brain barrier (BBB), and with different cytochrome p450 isoforms were particularly involved in drug elimination. Furthermore, drug-likeness depends mainly on the molecular features of the compounds and was compatible with ADMET in the body. Also, oral drug administration is a systematic route of delivering drug to pharmacologically active drugs, therefore; Oral bioavailability was assessed by evaluating the Veber rule, Egan rule, and Bayer oral PhysChem score, indicating reliable ADME properties approved as oral drug administration. Importantly, drug safety profiles were assessed by GSK 4/400 rule, Pfizer 3/75 rule, and phospholipidosis non-inducer, which are non-toxic profiles. Given the leads drug-likeness properties, further comprehensive pharmacological evaluations are recommended to hopefully ensure anticancer properties for effective clinical management of cancer.

**Table 4:** Physico-chemical properties, lipophilicity, solubility, pharmacokinetic, drug likeness and medicinal chemistry properties of the PI3K-α and PKC-η inhibitors.

Leads	Physicochemical properties	Lipophilicity	Water solubility	Pharmacokinetics	Drug likeness	Medicinal chemistry
**PI3K-α inhibitors**						
1	Formula: C32H40ClN3O5 Molecular weight: 582.13 g/mol Num. heavy atoms: 41 Num. arom. heavy atoms: 15 Fraction Csp3: 0.53 Num. rotatable bonds: 5 Num. H-bond acceptors: 5 Num. H-bond donors: 3 Molar Refractivity: 168.83 TPSA: 98.40 Å^2^	Log Po/w (iLOGP): 4.47 Log Po/w (XLOGP3): 4.66 Log Po/w (WLOGP): 4.69 Log Po/w (MLOGP): 3.06 Log Po/w (SILICOS-IT): 3.93 Consensus Log Po/w : 4.16	Log S (ESOL): -6.33 Solubility: 2.75e-04 mg/ml ; 4.72e-07 mol/l Class: Poorly soluble Log S (Ali): -6.45 Solubility: 2.05e-04 mg/ml ; 3.52e-07 mol/l Class: Poorly soluble Log S (SILICOS-IT): -6.40 Solubility: 2.30e-04 mg/ml ; 3.94e-07 mol/l Class: Poorly soluble	GI absorption: High BBB permeant: No P-gp substrate: No CYP1A2 inhibitor: No CYP2C19 inhibitor: No CYP2C9 inhibitor: No CYP2D6 inhibitor: No CYP3A4 inhibitor: No Log Kp (skin permeation): -6.54 cm/s	Lipinski: Yes; 1 violation: MW > 500 Ghose: No; 3 violations: MW > 480, MR > 130, #atoms > 70 Veber: Yes Egan: Yes Muegge: Yes Bioavailability Score: 0.55	PAINS: 0 alert Brenk: 0 alert Leadlikeness: No; 2 violations: MW > 350, XLOGP3 > 3.5 Synthetic accessibility: 4.99
2	Formula: C27H35N3O4 Molecular weight: 465.58 g/mol Num. heavy atoms: 34 Num. arom. heavy atoms: 12 Fraction Csp3: 0.48 Num. rotatable bonds: 8 Num. H-bond acceptors: 6 Num. H-bond donors: 1 Molar Refractivity: 140.50 TPSA : 75.13 Å^2^	Log Po/w (iLOGP): 4.24 Log Po/w (XLOGP3): 3.47 Log Po/w (WLOGP): 2.85 Log Po/w (MLOGP): 1.56 Log Po/w (SILICOS-IT): 3.60 Consensus Log Po/w : 3.14	Log S (ESOL): -4.65 Solubility: 1.05e-02 mg/ml ; 2.26e-05 mol/l Class: Moderately soluble Log S (Ali): -4.73 Solubility: 8.67e-03 mg/ml ; 1.86e-05 mol/l Class: Moderately soluble Log S (SILICOS-IT): -5.75 Solubility: 8.30e-04 mg/ml ; 1.78e-06 mol/l Class: Moderately soluble	GI absorption: High BBB permeant: Yes P-gp substrate: Yes CYP1A2 inhibitor: No CYP2C19 inhibitor: No CYP2C9 inhibitor: No CYP2D6 inhibitor: Yes CYP3A4 inhibitor: Yes Log K_p_ (skin permeation): -6.68 cm/s	Lipinski: Yes; 0 violation Ghose: No; 1 violation: MR > 130 Veber: Yes Egan: Yes Muegge: Yes Bioavailability Score: 0.55	PAINS: 0 alert Brenk: 0 alert Leadlikeness: No; 2 violations: MW > 350, Rotors > 7 Synthetic accessibility: 4.14
3	Formula: C26H31N5O3 Molecular weight: 461.56 g/mol Num. heavy atoms: 34 Num. arom. heavy atoms: 17 Fraction Csp3: 0.42 Num. rotatable bonds: 7 Num. H-bond acceptors: 6 Num. H-bond donors: 1 Molar Refractivity: 136.27 TPSA : 83.72 Å^2^	Log P_o/w_ (iLOGP): 2.88 Log P_o/w_ (XLOGP3): 1.79 Log P_o/w_ (WLOGP): 1.42 Log P_o/w_ (MLOGP): 1.27 Log P_o/w_ (SILICOS-IT): 2.61 Consensus Log P_o/w_ : 1.99	Log S (ESOL): -3.74 Solubility: 8.45e-02 mg/ml ; 1.83e-04 mol/l Class: Soluble Log S (Ali): -3.17 Solubility: 3.14e-01 mg/ml ; 6.81e-04 mol/l Class: Soluble Log S (SILICOS-IT): -5.91 Solubility: 5.73e-04 mg/ml ; 1.24e-06 mol/l Class: Moderately soluble	GI absorption: High BBB permeant: No P-gp substrate: Yes CYP1A2 inhibitor: No CYP2C19 inhibitor: No CYP2C9 inhibitor: Yes CYP2D6 inhibitor: Yes CYP3A4 inhibitor: Yes Log K_p_ (skin permeation): -7.84 cm/s	Lipinski: Yes; 0 violation Ghose: No; 1 violation: MR > 130 Veber: Yes Egan: Yes Muegge: Yes Bioavailability Score: 0.55	PAINS: 0 alert Brenk : 0 alert Leadlikeness; No; 1 violation: MW > 350 Synthetic accessibility: 4.21
Ppictilisilinib	Formula: C23H27N7O3S2 Molecular weight: 513.64 g/mol Num. heavy atoms: 35 Num. arom. heavy atoms: 18 Fraction Csp3: 0.43 Num. rotatable bonds: 5 Num. H-bond acceptors: 8 Num. H-bond donors: 1 Molar Refractivity: 147.70 TPSA: 144.17 Å^2^	Log P_o/w_ (iLOGP): 3.01 Log P_o/w_ (XLOGP3): 1.62 Log P_o/w_ (WLOGP): 1.93 Log P_o/w_ (MLOGP): 0.94 Log P_o/w_ (SILICOS-IT): 2.57 Consensus Log P_o/w_ : 2.01	Log S (ESOL) : -4.10 Solubility: 4.12e-02 mg/ml ; 8.02e-05 mol/l Class : Moderately soluble Log S (Ali): -4.26 Solubility: 2.82e-02 mg/ml ; 5.50e-05 mol/l Class : Moderately soluble Log S (SILICOS-IT) : -6.12 Solubility: 3.91e-04 mg/ml ; 7.60e-07 mol/l Class : Poorly soluble	GI absorption: Low BBB permeant : No P-gp substrate : Yes CYP1A2 inhibitor : No CYP2C19 inhibitor : Yes CYP2C9 inhibitor : Yes CYP2D6 inhibitor : Yes CYP3A4 inhibitor : Yes Log K_p_ (skin permeation) : -8.28 cm/s	Lipinski: Yes; 1 violation: MW > 500 Ghose : No; 2 violations: MW > 480, MR > 130 Veber : No; 1 violation: TPSA > 140 Egan : No; 1 violation: TPSA > 131.6 Muegge : Yes Bioavailability Score : 0.55	PAINS : 0 alert Brenk : 0 alert Leadlikeness : No; 1 violation: MW > 350 Synthetic accessibility : 3.95
**PKC-η inhibitors**						
1	Formula: C23H18N2O4S Molecular weight: 418.47 g/mol Num. heavy atoms: 30 Num. arom. heavy atoms: 21 Fraction Csp3: 0.13 Num. rotatable bonds: 3 Num. H-bond acceptors: 5 Num. H-bond donors: 2 Molar Refractivity: 119.78 TPSA : 111.13 Å^2^	Log P_o/w_ (iLOGP) : 3.05 Log P_o/w_ (XLOGP3) : 4.58 Log P_o/w_ (WLOGP) : 3.88 Log P_o/w_ (MLOGP) : 2.31 Log P_o/w_ (SILICOS-IT) : 4.50 Consensus Log P_o/w_ : 3.66	Log S (ESOL) : -5.64 Solubility: 9.59e-04 mg/ml ; 2.29e-06 mol/l Class : Moderately soluble Log S (Ali) : -6.64 Solubility: 9.64e-05 mg/ml ; 2.30e-07 mol/l Class : Poorly soluble Log S (SILICOS-IT) : -6.62 Solubility: 1.02e-04 mg/ml ; 2.43e-07 mol/l Class : Poorly soluble	GI absorption : High BBB permeant : No P-gp substrate : No CYP1A2 inhibitor : No CYP2C19 inhibitor : Yes CYP2C9 inhibitor : Yes CYP2D6 inhibitor : No CYP3A4 inhibitor : Yes Log K_p_ (skin permeation) : -5.60 cm/s	Lipinski : Yes; 0 violation Ghose : Yes Veber : Yes Egan : Yes Muegge : Yes Bioavailability Score : 0.55	PAINS : 0 alert Brenk : 0 alert Leadlikeness : No; 2 violations: MW > 350, XLOGP3 > 3.5 Synthetic accessibility : 3.30
2	Formula: C21H21N3O3S Molecular weight: 395.47 g/mol Num. heavy atoms: 28 Num. arom. heavy atoms: 15 Fraction Csp3: 0.33 Num. rotatable bonds: 3 Num. H-bond acceptors: 5 Num. H-bond donors: 2 Molar Refractivity: 112.91 TPSA : 116.92 Å^2^	Log P_o/w_ (iLOGP) : 3.04 Log P_o/w_ (XLOGP3) : 2.52 Log P_o/w_ (WLOGP) : 2.74 Log P_o/w_ (MLOGP) : 1.83 Log P_o/w_ (SILICOS-IT) : 3.89 Consensus Log P_o/w_ : 2.80	Log S (ESOL): -4.08 Solubility: 3.30e-02 mg/ml ; 8.36e-05 mol/l Class : Moderately soluble Log S (Ali): -4.62 Solubility: 9.45e-03 mg/ml ; 2.39e-05 mol/l Class : Moderately soluble Log S (SILICOS-IT) : -5.43 Solubility: 1.46e-03 mg/ml ; 3.70e-06 mol/l Class : Moderately soluble	GI absorption: High BBB permeant : No P-gp substrate : Yes CYP1A2 inhibitor : Yes CYP2C19 inhibitor : Yes CYP2C9 inhibitor : Yes CYP2D6 inhibitor : Yes CYP3A4 inhibitor : Yes Log K_p_ (skin permeation) : -6.92 cm/s	Lipinski : Yes; 0 violation Ghose : Yes Veber : Yes Egan : Yes Muegge : Yes Bioavailability Score : 0.55	PAINS : 0 alert Brenk : 0 alert Leadlikeness : No; 1 violation: MW > 350 Synthetic accessibility : 3.27
3	Formula: C22H20N2O5 Molecular weight: 392.40 g/mol Num. heavy atoms: 29 Num. arom. heavy atoms: 18 Fraction Csp3: 0.18 Num. rotatable bonds: 3 Num. H-bond acceptors: 6 Num. H-bond donors: 3 Molar Refractivity: 111.39 TPSA : 103.12 Å^2^	Log P_o/w_ (iLOGP) : 1.90 Log P_o/w_ (XLOGP3) : 3.36 Log P_o/w_ (WLOGP) : 2.68 Log P_o/w_ (MLOGP) : 1.58 Log P_o/w_ (SILICOS-IT) : 2.90 Consensus Log P_o/w_ : 2.48	Log S (ESOL) : -4.65 Solubility: 8.76e-03 mg/ml ; 2.23e-05 mol/l Class : Moderately soluble Log S (Ali) : -5.20 Solubility: 2.46e-03 mg/ml ; 6.26e-06 mol/l Class : Moderately soluble Log S (SILICOS-IT) : -5.50 Solubility: 1.23e-03 mg/ml ; 3.13e-06 mol/l Class : Moderately soluble	GI absorption: High BBB permeant : No P-gp substrate : Yes CYP1A2 inhibitor : No CYP2C19 inhibitor : No CYP2C9 inhibitor : Yes CYP2D6 inhibitor : Yes CYP3A4 inhibitor : Yes Log K_p_ (skin permeation) : -6.31 cm/s	Lipinski : Yes; 0 violation Ghose : Yes Veber : Yes Egan : Yes Muegge : Yes Bioavailability Score : 0.55	PAINS: 0 alert Brenk : 0 alert Leadlikeness: No; 1 violation: MW > 350 Synthetic accessibility : 3.18
4	Formula: C22H21N3O4 Molecular weight: 391.42 g/mol Num. heavy atoms: 29 Num. arom. heavy atoms: 18 Fraction Csp3: 0.23 Num. rotatable bonds: 3 Num. H-bond acceptors: 6 Num. H-bond donors: 2 Molar refractivity: 112.12 TPSA : 95.78 Å^2^	Log P_o/w_ (iLOGP) : 2.41 Log P_o/w_ (XLOGP3) : 2.23 Log P_o/w_ (WLOGP) : 2.67 Log P_o/w_ (MLOGP) : 0.91 Log P_o/w_ (SILICOS-IT) : 3.34 Consensus Log P_o/w_ : 2.31	Log S (ESOL) : -3.93 Solubility: 4.57e-02 mg/ml ; 1.17e-04 mol/l Class : Soluble Log S (Ali) : -3.88 Solubility: 5.20e-02 mg/ml ; 1.33e-04 mol/l Class : Soluble Log S (SILICOS-IT) : -6.10 Solubility: 3.14e-04 mg/ml ; 8.02e-07 mol/l Class : Poorly soluble	GI absorption : High BBB permeant : No P-gp substrate : Yes CYP1A2 inhibitor : No CYP2C19 inhibitor : No CYP2C9 inhibitor : Yes CYP2D6 inhibitor : No CYP3A4 inhibitor : Yes Log K_p_ (skin permeation) : -7.10 cm/s	Lipinski : Yes; 0 violation Ghose : Yes Veber : Yes Egan : Yes Muegge : Yes Bioavailability Score : 0.55	PAINS : 0 alert Brenk : 0 alert Leadlikeness : No; 1 violation: MW > 350 Synthetic accessibility : 3.35
5	Formula: C23H24N4O3 Molecular weight: 404.46 g/mol Num. heavy atoms: 30 Num. arom. heavy atoms: 18 Fraction Csp3: 0.26 Num. rotatable bonds: 6 Num. H-bond acceptors: 5 Num. H-bond donors: 2 Molar Refractivity: 121.13 TPSA : 89.79 Å^2^	Log P_o/w_ (iLOGP) : 2.15 Log P_o/w_ (XLOGP3) : 2.14 Log P_o/w_ (WLOGP) : 2.00 Log P_o/w_ (MLOGP) : 1.26 Log P_o/w_ (SILICOS-IT) : 2.36 Consensus Log P_o/w_ : 1.98	Log S (ESOL) : -3.74 Solubility: 7.29e-02 mg/ml ; 1.80e-04 mol/l Class : Soluble Log S (Ali) : -3.66 Solubility: 8.90e-02 mg/ml ; 2.20e-04 mol/l Class : Soluble Log S (SILICOS-IT) : -5.64 Solubility: 9.31e−04 mg/ml ; 2.30e−06 mol/l Class : Moderately soluble	GI absorption : High BBB permeant : No P-gp substrate : Yes CYP1A2 inhibitor : No CYP2C19 inhibitor: No CYP2C9 inhibitor : No CYP2D6 inhibitor : Yes CYP3A4 inhibitor : Yes Log K_p_ (skin permeation) : -7.25 cm/s	Lipinski : Yes; 0 violation Ghose : Yes Veber: Yes Egan : Yes Muegge : Yes Bioavailability Score : 0.55	PAINS : 0 alert Brenk : 0 alert Leadlikeness : No; 1 violation: MW > 350 Synthetic accessibility : 3.48
6	Formula: C23H24N2O4 Molecular weight: 392.45 g/mol Num. heavy atoms: 29 Num. arom. heavy atoms: 18 Fraction Csp3: 0.26 Num. rotatable bonds: 4 Num. H-bond acceptors: 6 Num. H-bond donors: 2 Molar Refractivity: 115.43 TPSA : 75.05 Å^2^	Log P_o/w_ (iLOGP) : 3.57 Log P_o/w_ (XLOGP3) : 3.21 Log P_o/w_ (WLOGP) : 3.19 Log P_o/w_ (MLOGP) : 1.59 Log P_o/w_ (SILICOS-IT) : 3.90 Consensus Log P_o/w_ : 3.09	Log S (ESOL) : -4.49 Solubility: 1.27e-02 mg/ml ; 3.23e-05 mol/l Class : Moderately soluble Log S (Ali) : -4.46 Solubility: 1.37e-02 mg/ml ; 3.48e-05 mol/l Class : Moderately soluble Log S (SILICOS-IT) : -6.66 Solubility: 8.51e-05 mg/ml ; 2.17e-07 mol/l Class : Poorly soluble	GI absorption : High BBB permeant : Yes P-gp substrate : Yes CYP1A2 inhibitor : No CYP2C19 inhibitor : No CYP2C9 inhibitor : No CYP2D6 inhibitor : Yes CYP3A4 inhibitor : Yes Log K_p_ (skin permeation) : -6.41 cm/s	Lipinski : Yes; 0 violation Ghose : Yes Veber : Yes Egan : Yes Muegge : Yes Bioavailability Score : 0.55	PAINS: 0 alert Brenk : 0 alert Leadlikeness : No; 1 violation: MW > 350 Synthetic accessibility : 3.42
7	Formula: C19H20ClN3O4 Molecular weight: 389.83 g/mol Num. heavy atoms: 27 Num. arom. heavy atoms: 12 Fraction Csp3: 0.37 Num. rotatable bonds: 3 Num. H-bond acceptors: 6 Num. H-bond donors: 3 Molar Refractivity: 107.67 TPSA : 94.92 Å^2^	Log P_o/w_ (iLOGP) : 2.86 Log P_o/w_ (XLOGP3) : 0.98 Log P_o/w_ (WLOGP) : 0.64 Log P_o/w_ (MLOGP) : 0.42 Log P_o/w_ (SILICOS-IT) : 2.01 Consensus Log P_o/w_ : 1.38	Log S (ESOL) : -3.01 Solubility: 3.85e-01 mg/ml ; 9.88e-04 mol/l Class : Soluble Log S (Ali) : -2.56 Solubility: 1.07e + 00 mg/ml ; 2.74e-03 mol/l Class : Soluble Log S (SILICOS-IT) : -4.49 Solubility: 1.25e-02 mg/ml ; 3.21e-05 mol/l Class : Moderately soluble	GI absorption : High BBB permeant : No P-gp substrate : Yes CYP1A2 inhibitor : No CYP2C19 inhibitor : No CYP2C9 inhibitor : No CYP2D6 inhibitor : Yes CYP3A4 inhibitor : No Log K_p_ (skin permeation) : -7.98 cm/s	Lipinski : Yes; 0 violation Ghose : Yes Veber : Yes Egan : Yes Muegge : Yes Bioavailability Score : 0.55	PAINS : 0 alert Brenk : 0 alert Leadlikeness : No; 1 violation: MW > 350 Synthetic accessibility : 3.77
Staurosporine	Formula: C28H26N4O3 Molecular weight: 466.53 g/mol Num. heavy atoms: 35 Num. arom. heavy atoms: 15 Fraction Csp3: 0.32 Num. rotatable bonds: 2 Num. H-bond acceptors: 4 Num. H-bond donors: 2 Molar Refractivity: 139.27 TPSA : 67.76 Å^2^	Log P_o/w_ (iLOGP) : 3.26 Log P_o/w_ (XLOGP3) : 1.97 Log P_o/w_ (WLOGP) : 0.70 Log P_o/w_ (MLOGP) : 1.87 Log P_o/w_ (SILICOS-IT) : 2.01 Consensus Log P_o/w_ : 1.96	Log S (ESOL) : -4.16 Solubility: 3.24e-02 mg/ml ; 6.94e-05 mol/l Class : Moderately soluble Log S (Ali) : -3.02 Solubility: 4.47e-01 mg/ml ; 9.58e-04 mol/l Class : Soluble Log S (SILICOS-IT) : -6.63 Solubility: 1.10e-04 mg/ml ; 2.37e-07 mol/l Class : Poorly soluble	GI absorption : High BBB permeant : No P-gp substrate : Yes CYP1A2 inhibitor : Yes CYP2C19 inhibitor : Yes CYP2C9 inhibitor : No CYP2D6 inhibitor : Yes CYP3A4 inhibitor : Yes Log K_p_ (skin permeation) : -7.75 cm/s	Lipinski : Yes; 0 violation Ghose : No; 1 violation: MR > 130 Veber : Yes Egan : Yes Muegge : No; 1 violation: #rings > 7 Bioavailability Score : 0.55	PAINS: 1 alert: ene_five_het_C Brenk : 0 alert Leadlikeness : No; 1 violation: MW > 350 Synthetic accessibility : 6.01

## Materials and methods

### Protein preparation

Crystal structures of PKC-η (PDB ID: 3TXO)^43^, HRas-P21 (PDB ID: 121P)^44^, AKT-1 (PDB ID: 3CQW)^45^, Ras (PDB ID: 1LFD)^46^, PI3K-α (PDB ID: 4A55)^47^, MEKK3 (PDB ID: 2PPH)^48^, NFκB-P52 (PDB ID: 1A3Q)^49^, MEKK2b (PDB ID: 2CU1)^50^ and TRAF2 (PDB ID: 3M06)^51^ were retrieved from Protein Data Bank (PDB). However, identification of missing regions in the crystal structures was accomplished with SEQATOMs (Fig. S1; Table S1)^52^. Five missing regions have been found in crystal structure of PI3K-α (4A55): region-I (M1-R32), region-II (R309-S323), region-III (V409-C420), region-IV (V500-N526) and region-V (C862-Q871), respectively. These missing regions were built based on 5SXA (PDB ID)^53^. The missing regions were found away from the active pocket. A missing region (C506-G516) was identified in crystal structure of PKC-η and was built from PDB ID: 3TXO. Subsequently, homology modeling was carried out for missing residues using MOE. Water molecules, cofactors, and bound ligands were extracted and energy was minimized with the molecular operating environment (MOE) using the following methods and parameters. Hydrogens were added and the gradient was set to 0.00001. The force field of the MMFF94x was set up with the cut off value enabled from 8 to 10, the solvation was set to distance mode, the exterior was set to 8, the dielectric was set to 1, and furthermore, the partial charges have been fixed for the required calculations. After energy minimization, the proteins were successfully utilized for further studies.

### Pocket volumetric analysis

Binding pockets, size, shape, key residues, and pocket opening of drug targets were analyzed using a CASTp (Computed Atlas of Surface Topography of proteins) server^54^.

### Ligand preparation

The 3D atomic coordinates of the dietary agents were retrieved from PubChem. In addition, the molecular formulas, molecular weights, and IUPAC structure identifiers (InChI and standard InChIKey) were successfully retrieved, along with a core library consists of 735,000 diverse scaffolds available in ChemBridge (https://www.chembridge.com/index.php). Furthermore, to avoid errors in compound structures, for instance, single bonds, protonation, disordered bond lengths, tautomers, ionization states, and explicit counter ions, in this connection, the database was launched into MOE to remove faulty structures by washing. Energy minimization was achieved with 3D optimization of small molecules and the addition of hydrogens and atomic partial charges with MMFF94x forcefield^55^. Finally, the refined small molecules were examined in subsequent studies.

### Molecular docking

Molecular docking studies using Autodock Vina 4.0 with a PyRx interface have been conducted between dietary agents and cancer targets^56^. The docking parameters were set as follows: The Lamarckian genetic algorithm was used as the docking program by setting the window size 10.0^57^, and the number of individuals in the population was set to 150. The maximum number of energy evaluations and the maximum number of generations was set to 25,000 and 27,000. For the survival of next-generation, the top individual was set to 1. The gene mutation rate was set to 0.02. The crossover rate was set to 0.8, and the cauchy beta was 1.0. Accordingly, the grid was set according to the binding pocket and the exhaustiveness was set to 8. After docking simulations, the binding energy cutoff was set between 3.0 and 11.0 kcal/mol. Bond angles, bond lengths, and hydrogen bonding interactions were analyzed using PyMOL^58^ and electrostatic bond distance was set to between 3.0–5.0 Å, the hydrophobic bond was set to range 3.0–5.0 Å, and the hydrogen bond was set to between 2.8 and 4.0 Å.

### Pharmacophore modeling and virtual screening

Crystal structures of PI3K-α and PKC-η co-crystalized with various ligands resolved at high resolution were retrieved from PDB. However, the protein ligand interaction fingerprints (PLIF) method is used to summarize the interaction in the complexes, the PLIF tool is available in MOE. Additionally, it also generates a set of pharmacophore query features from the annotation point of the receptor-ligand complex. These pharmacophore features include a significant group or group of H-donors (Don), H-acceptors (Acc), aromatic centers (Aro), Hydrophobic (Hyd), donor and acceptor (Don & Acc), R-groups and bioisosteres, respectively. The generated pharmacophore query contains a set of constraints and features. In MOE, potential setup was used to generate forcefield parameters. The force field was set to amber10: EHT and the solvation was set to R-field. Numerous molecular interactions such as van der Waals forces, electrostatic, and restrains have been enabled. Hydrogens and partial chargers were added. The receptor strength was tethered to 5000 to keep the receptor rigid and this allows 3D protonation and removes water molecules from the receptor or the ligand within 4.5 Å. The scheme was set to EHT to construct the pharmacophore query from the ligand annotation points. The annotation points were indicated in various colors, green for hydrophobic, purple for hydrogen bond donor, cyan for hydrogen bond acceptor, and orange for aromatic. Subsequently, the generated pharmacophore query was verified and improved by repeating the pharmacophoric feature search iteratively by changing the features. To obtain potential leads, the pharmacophore features were reduced according to catalytic residues in the cavity for selective binding and orientations. A virtual screening (VS) is important approach in computer-aided drug design to screen a huge chemical library^59^. In this study, pharmacophore-based virtual screening was used to screen a refined ChemBridge database using a modified and validated pharmacophore queries. Consequently, retrieved hits with minimal binding energy conformations were saved in a separate MOE database using the conformation import method. Since then, the conformers of each ligand have been filtered through recognizable pharmacophoric features, which can be considered as a potential virtual hit. Therefore, the best hits in the developed pharmacophore model were identified, mapped and further assessed with docking simulation.

### Born interaction energies and binding affinities

The binding affinity of the lead molecules was calculated using general born-volume integral (MM/GBVI) implicit solvent methods implemented in MOE^60–63^. According to the Eq. (1), the interactions energy (IE) is defined as the energy difference between the enzyme–substrate complex (E–S) and individual enzyme (E) and substrate (S).

1 $${\text{IE }} = {\text{ E}}_{{{\text{E}} - {\text{S}}}} - \, \left( {{\text{E}}_{{\text{E}}} + {\text{ E}}_{{\text{S}}} } \right)$$

Born interaction energy describes non-bonded contacts between the ligand and the protein by means of molecular interactions such as van der Waals, coulomb electrostatic, and solvent interactions. The strain energies and solvent molecules of the ligand and the protein were ignored during binding affinity evaluations. For the binding affinity calculations, the London scoring method was represented in kcal mol^−1^ units. To discourage gross movements, the active site residues and the ligand molecules were kept flexible whereas the active site was kept rigid to tether restraints. Furthermore, the binding affinity of the protein–ligand complex was calculated after the completion of energy minimization.

### Drug-likeness and toxicity property predictions

Lipinski rule of five such as molecular weight (< 500), H-bond acceptor (< 10), H-bond donor (< 5) and cLogP (< 5), toxic properties: mutagenic, tumorigenic, irritant and reproductive effects and ADMET (absorption, distribution, metabolism, excretion, and transport) properties were assessed using the FAF-Drugs server and the Swiss-ADME^64, 65^.

## Conclusion

Dietary compounds have potent anticancer properties and play a pivotal role in the prevention or treatment of cancer; however, the design of novel potent anticancer drugs require a specific molecular mechanism of the dietary compounds. In the current study, several dietary compounds have been subjected to docking studies against cancer therapeutic targets unveiled that naturally occurring dietary agents—silibinin, flavopiridol, oleandrin, ursolic acid, α-boswellic acid, β-boswellic acid, triterpenoid, guggulsterone, and oleanolic acid have been shown to have broad-spectrum anticancer properties by exhibiting inhibition against different cell signaling pathways. Importantly, silibinin and flavopiridol have significantly demonstrated broad-spectrum anticancer activity by targeting numerous drug targets—PI3K-α, PKC-η, H-Ras, and Ras. Interestingly, flavopiridol was embedded in the pockets of PI3K-α and PKC-η as bound crystal inhibitors and described critical interactions with hotspot residues. This prompted the designing of isoform inhibitors based on potent scaffold of various PI3K-α and PKC-η inhibitors using complex-based pharmacophore modeling. Finally, the top three optimistic lead compounds for PI3K-α, while seven for PKC-η from the top ligands were sorted and examined for important interactions with key residues. In addition, drug safety profiles, Lipinski rule, ADMET, pharmacokinetics, drug-likeness and toxic studies proposed for further validations by scaffold chemical synthesis and therapeutic evaluations. Accordingly, the drug targets have been identified for future research, which can be considered with its dietary agents to develop novel therapeutic approaches with better efficacy, specificity, and effective treatment of cancer pathogenesis without any side effects.

## Supplementary Information

Supplementary Information.
