# Supplementary material for: Molecular insight into isoform specific inhibition of PI3K-α and PKC-η with dietary agents through an ensemble pharmacophore and docking studies
Source: Sci Rep. 2021 Jun 9;11:12150. doi: 10.1038/s41598-021-90287-3 (PMC8190100; doi:10.1038/s41598-021-90287-3)
Supplement: Supplementary file 1 — Supplementary Information. [file 41598_2021_90287_MOESM1_ESM.pdf]

Supporting information

Molecular insight into isoform specific inhibition of PI3K-α and PKC-η with dietary agents through an ensemble pharmacophore and docking studies

Baki Vijaya Bhaskar[1]*, Aluru Rammohan[2], Tirumalasetty Munichandra Babu[3], Gui Yu Zheng[1], Weibin Chen[1], Rajendra Wudayagiri[4], Grigory V. Zyryanov[2], Wei Gu[1]*

[1]Department of Pathophysiology, The Key Immunopathology Laboratory of Guangdong Province, Shantou University Medical College, Shantou, Guangdong, China-515031.
[2]Department of organic and biomolecular chemistry, Ural Federal University, Ekaterinburg 620002, Russia.
[3]Department of Physiology, Shantou University Medical College, Shantou, Guangdong, China-515031.
[4]Department of Zoology, Sri Venkateswara University, Tirupati, Andhra Pradhesh-517502.

*Correspondence: vijaybio08@gmail.com, weigu@stu.edu.cn

FIGURE

a). **b).**

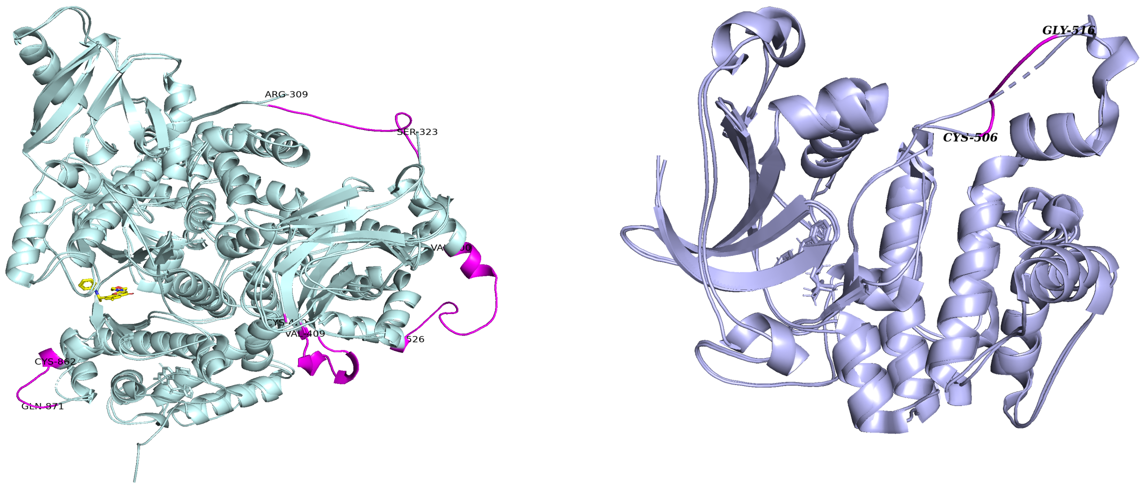

Figure S1. Overlays of modeled structures of **a).** PI3K-α and **b).** PKC-η with missing regions of crystal structures. Missing regions are marked in magenta with labeling.

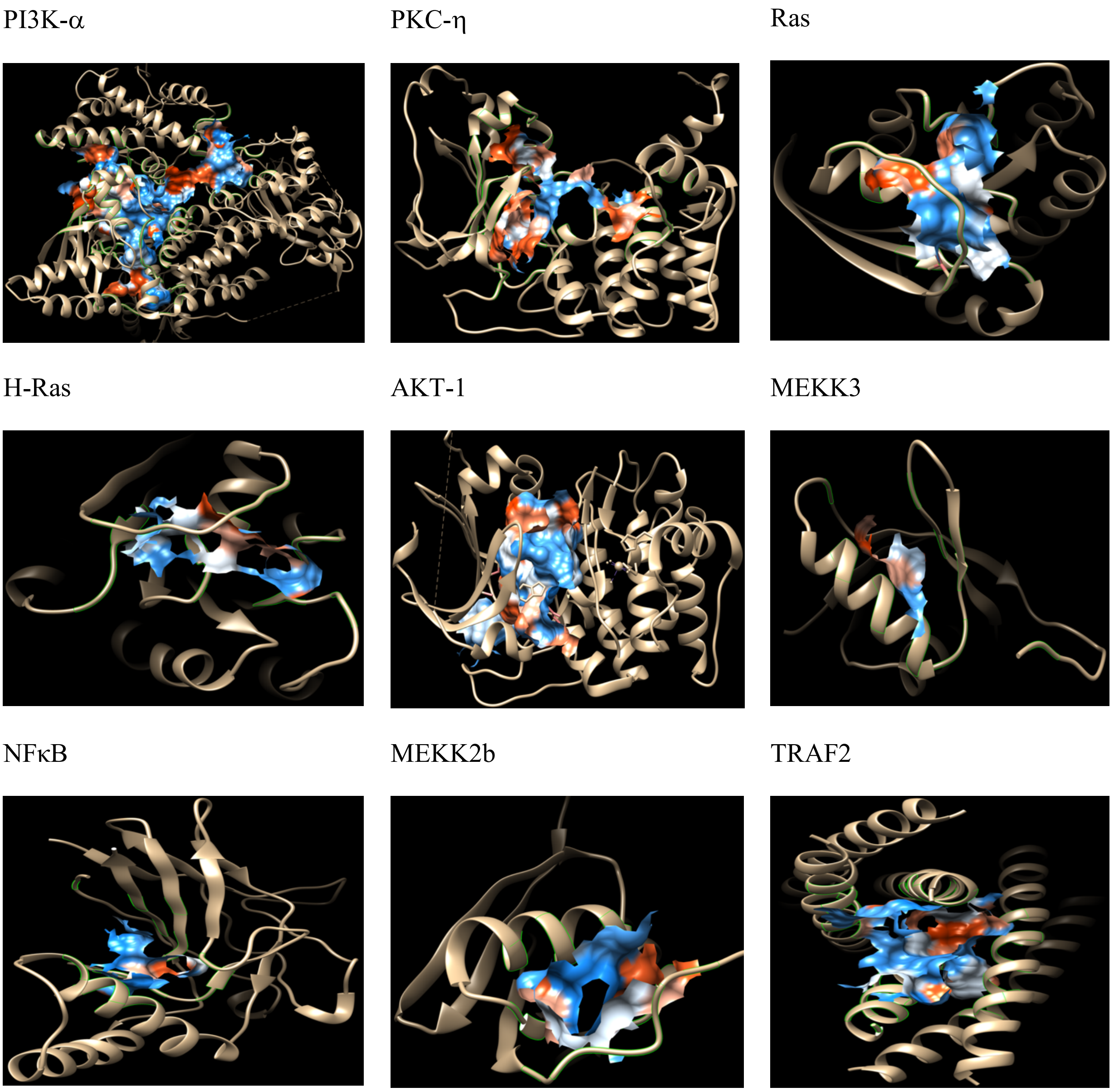

Figure S2. The shapes of the binding pocket cavity and surface area (SA) of molecular targets were analyzed using CASTp Server.

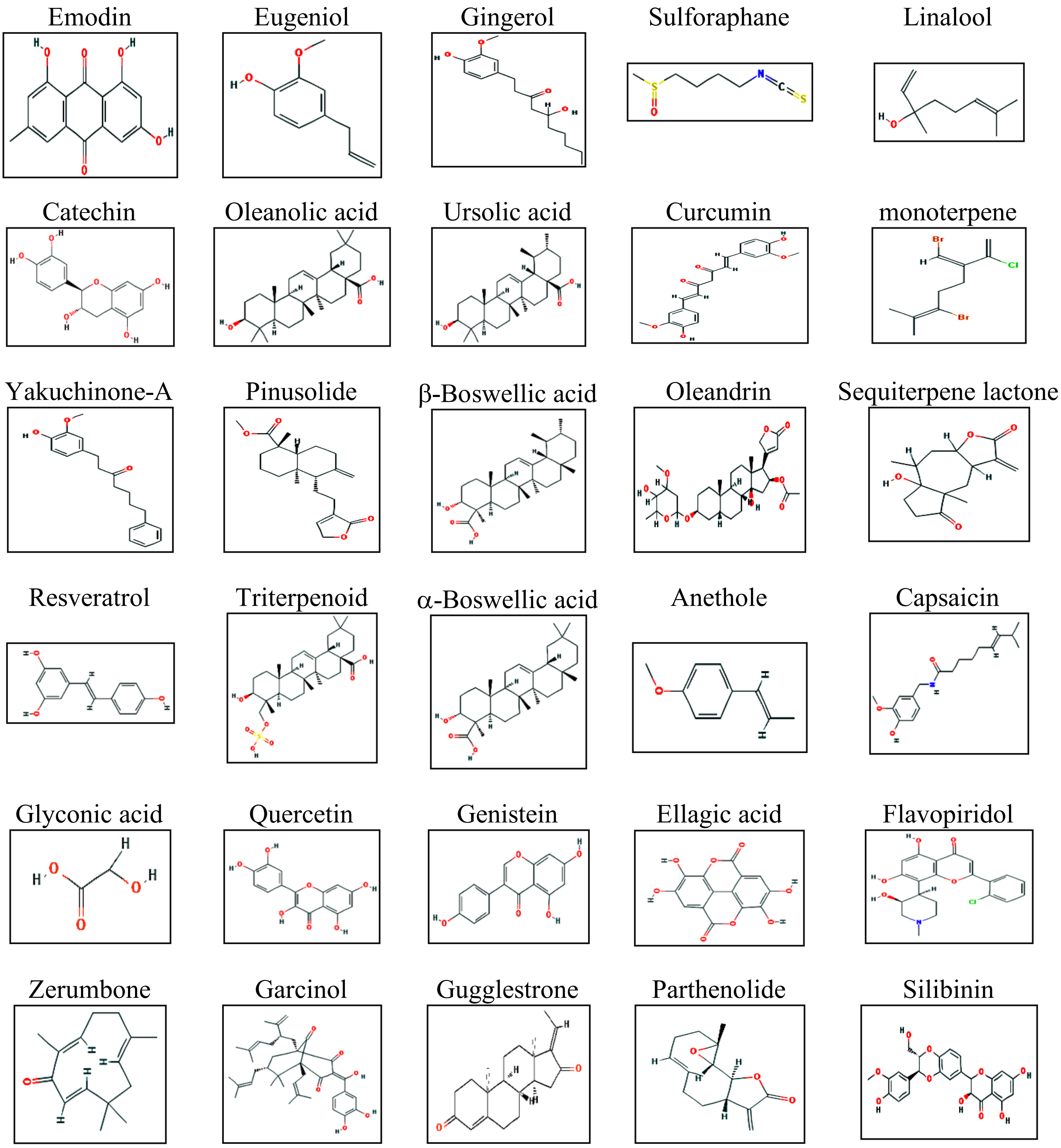

Figure S3. 2D structures of dietary compounds were retrieved from PubChem.

a).

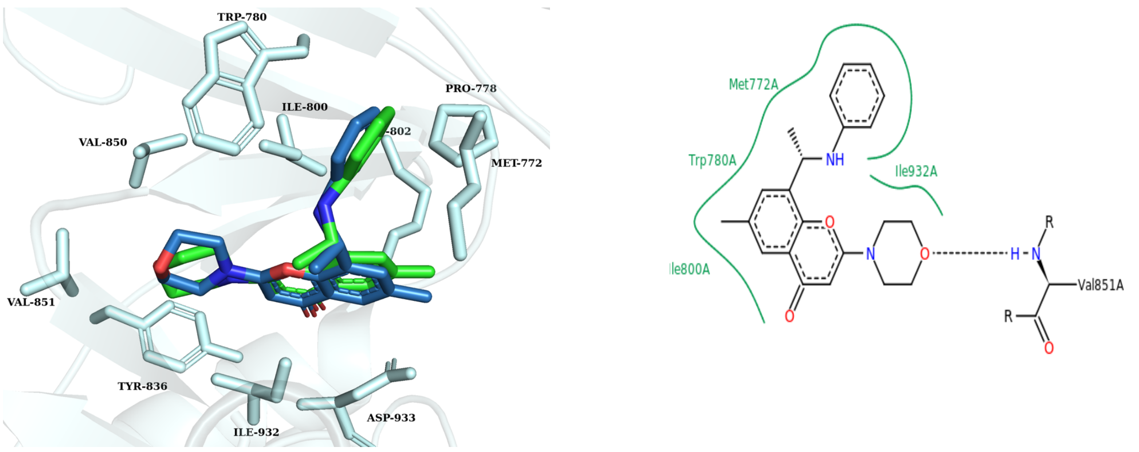

b).

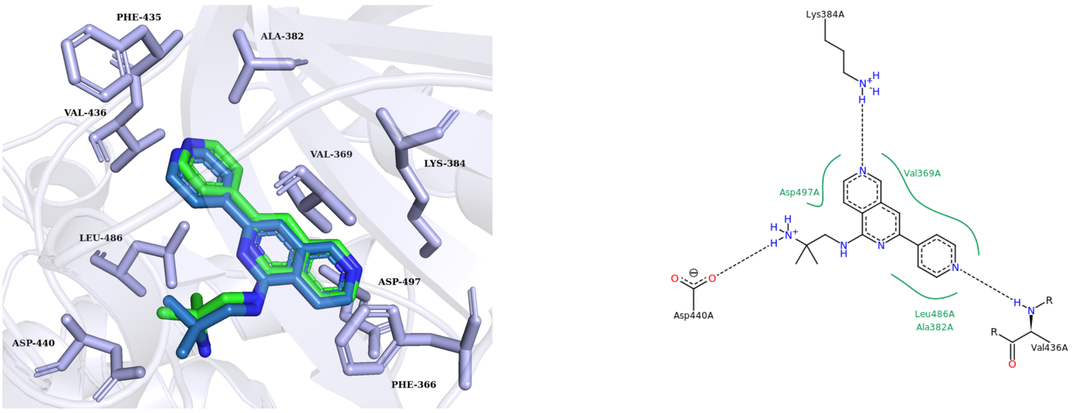

Figure S4. a). Overlays of crystal ligand PIK-108 (green) and docked pose in the ATP binding pocket of PI3K-α (p110α) and b). Overlays of cocrystal ligand 07U (green) and docked pose (blue) in the binding pocket of PKC-η.

Target	Compounds				
PI3K-α	Silibinin	Flavopiridol	Oleandrin	Ursolic acid	α-Boswellic acid
	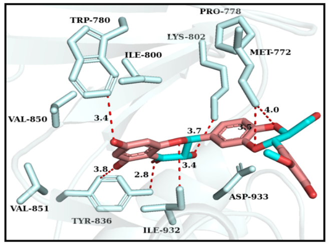	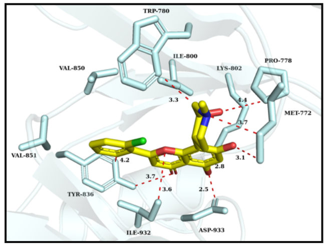	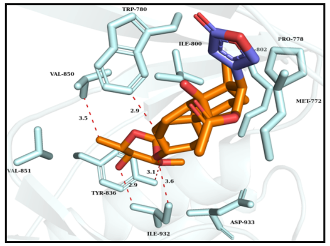	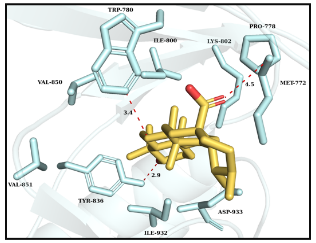	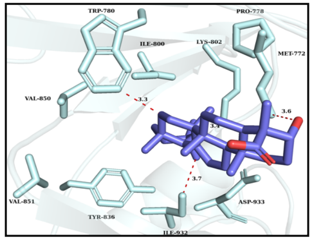
	β-Boswellic acid	Triterpinoid	Gugglesterone	Oleanolic acid	
	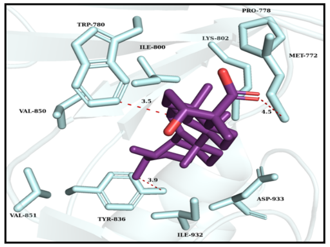	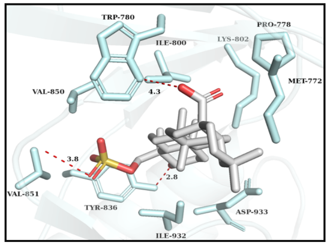	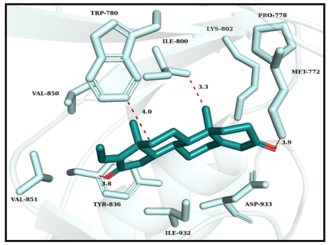	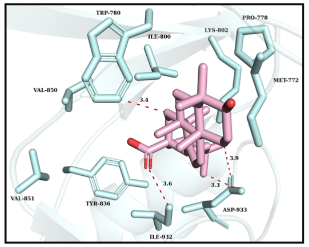	
PKC-η	Silibinin	Flavopiridol	Oleandrin	Ursolic acid	Emodin
	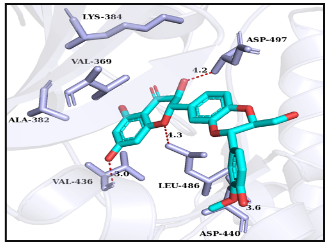	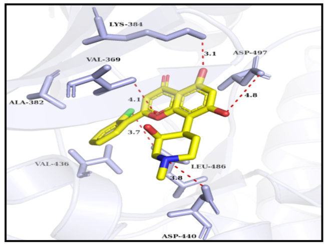	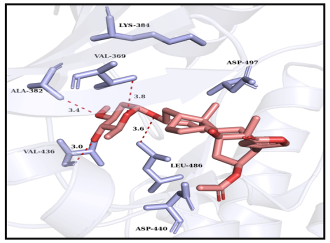	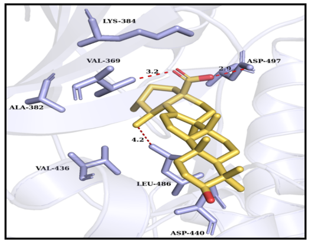	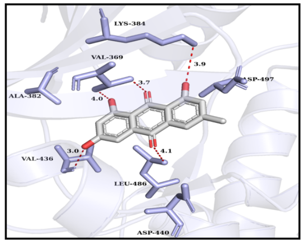
	Ellagic acid				

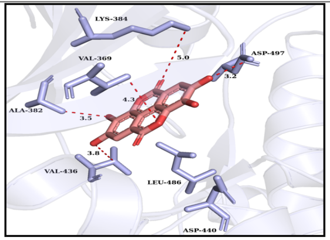

Ras

Silibinin

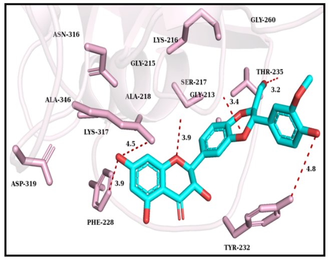

Flavopiridol

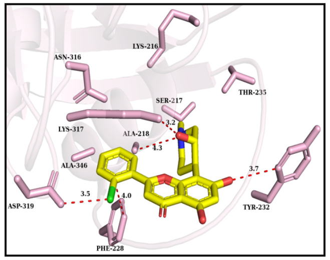

Oleandrin

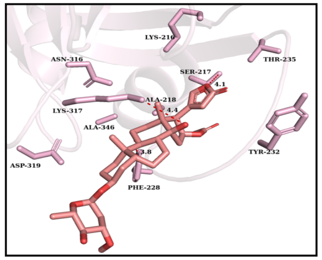

α-Boswellic acid

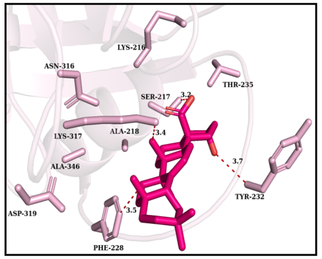

H-Ras

Silibinin

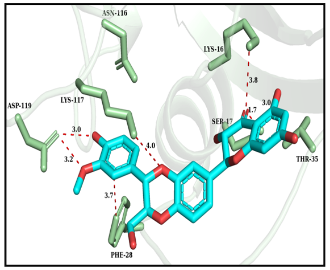

Figure S5. 3D orientation and binding interactions of the best docked dietary agents with active site residues of PI3K-α, PKC-η, Ras and H-Ras. Proteins are shown in the cartoon and key residues are represented sticks with labelling in the binding cavity. Binding interactions are indicated in red dotted lines with distances (Å).

Target	Dietary compound				
PI3K-α and crystal ligand PIK-108 (green)	Silibinin 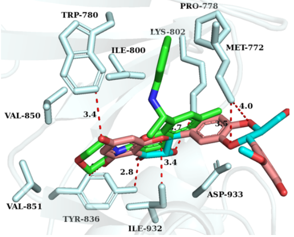	Flavopiridol 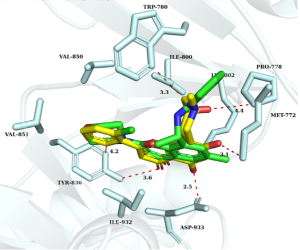	Oleandrin 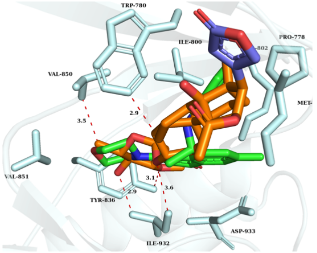	Ursolic acid 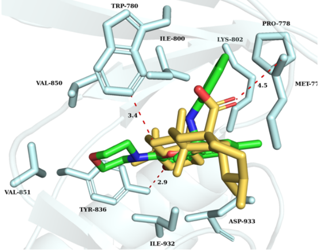	α-Boswellic acid 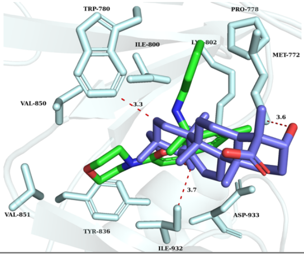
	β-Boswellic acid 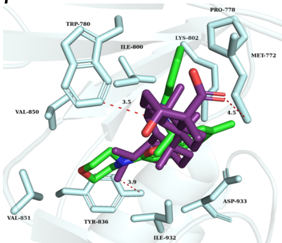	Triterpinoid 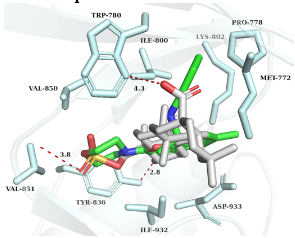	Gugglesterone 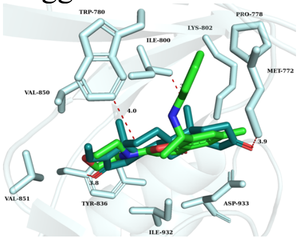	Oleanolic acid 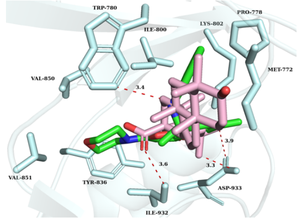	
PKC-η and crystal ligand naphthyridine (green)	Silibinin 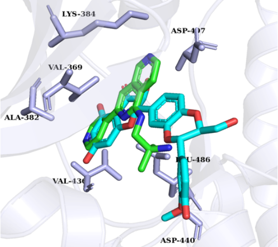	Flavopiridol 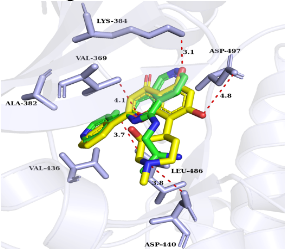	Oleandrin 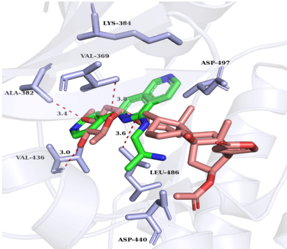	Ursolic acid 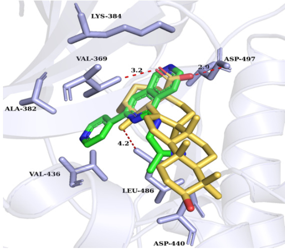	Emodin 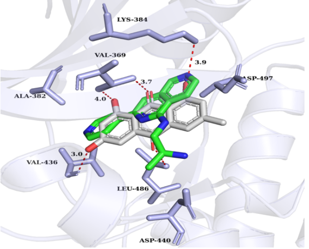

Ellagic acid

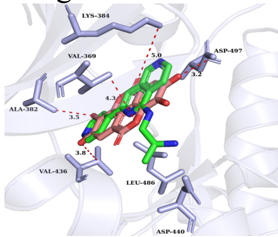

Ras and ATP

Silibinin

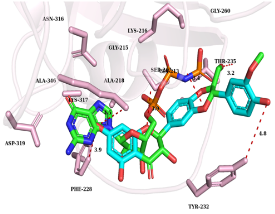

Flavopiridol

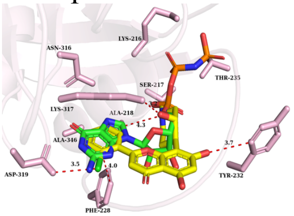

Oleandrin

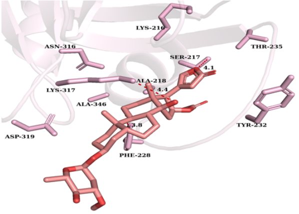

α-Boswellic acid

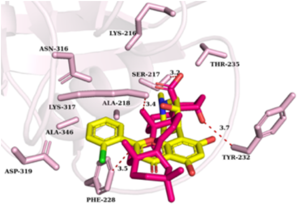

H-Ras and ATP

Silibinin and ATP

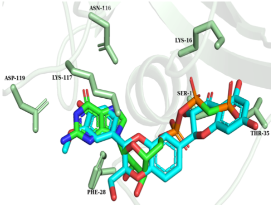

Figure S6. Overlay of the best docked dietary agents with reference ligands in binding pockets of cancer drug targets.

A.

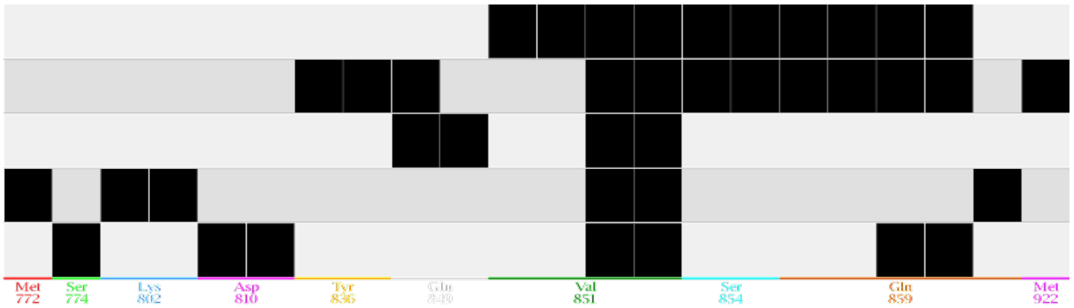

B.

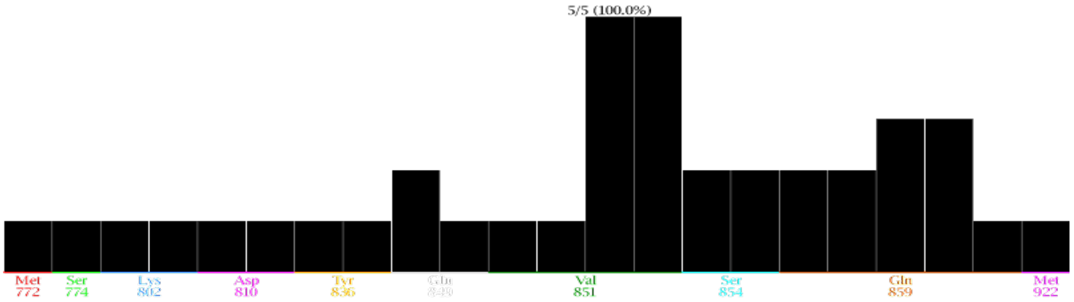

C.

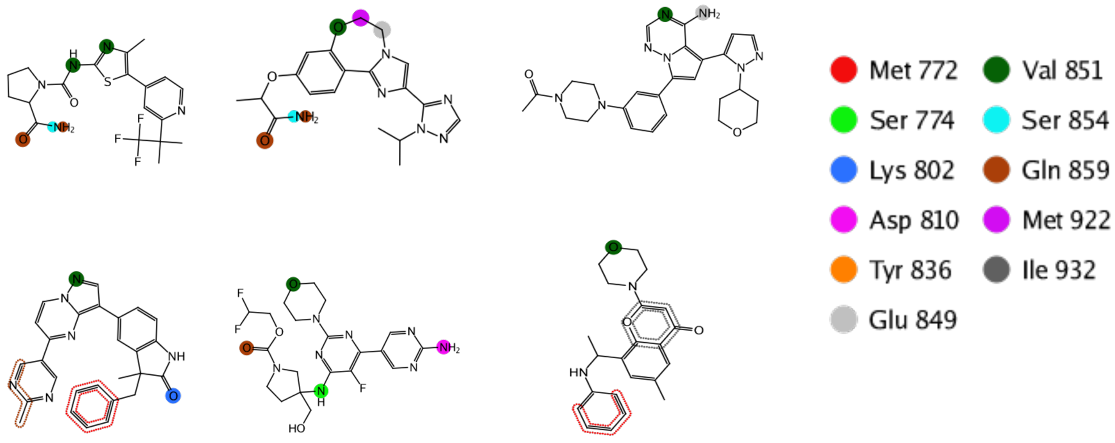

Figure S7. The PLIF computed the interactions between the PI3K-α-ligand complexes. **A).** The barcode representation of fingerprint of the PI3K-α-ligand complexes: The x-axis displays a three-letter code of the key residues and the y-axis shows the number of PI3K-α-ligand complexes. **B).** Population mode refers to the histogram of fingerprint of the PI3K-α-ligand complexes which showing the number of ligands with which each residue interacts. **C).** This display 2D depictions of the ligands. The colour highlights of the ligands correspond to the interactions shown in the barcodes and population modes. Notices that all interactions with a single protein residue will have the same colour in all the display modes.

A.

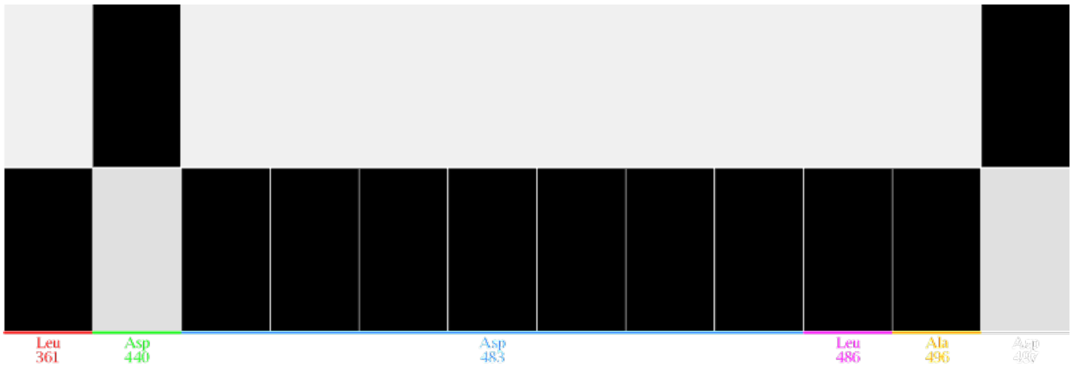

B.

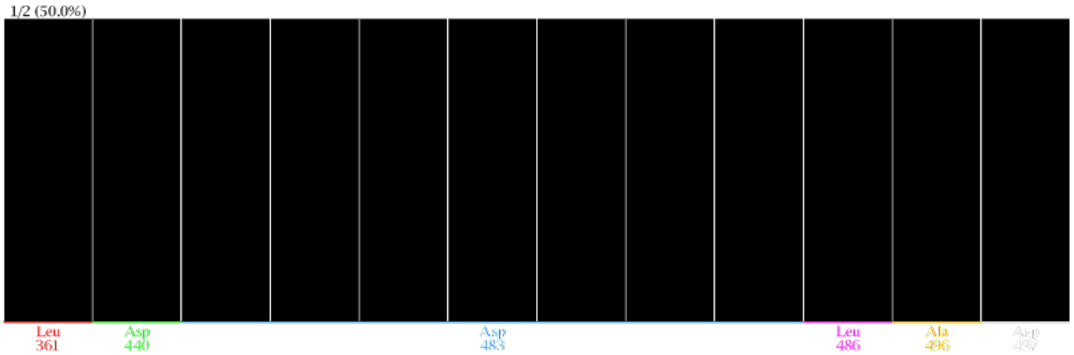

C.

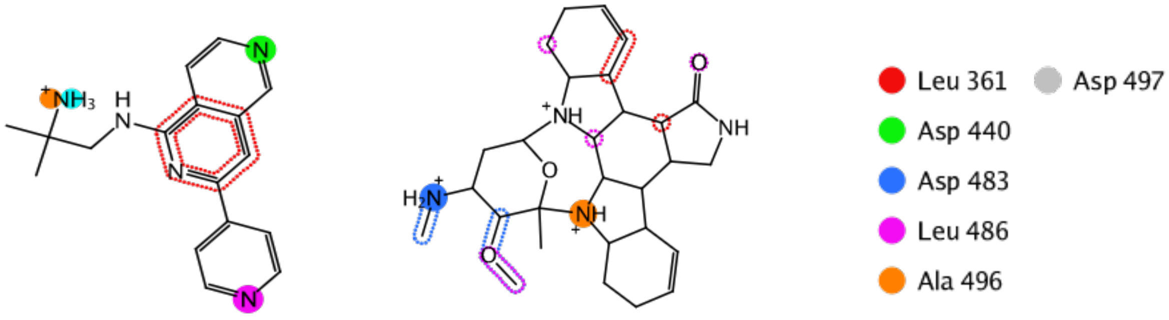

Figure S8. The PLIF computed the interactions between the PKC-η-ligand complexes. **A).** The barcode representation of fingerprint of the PKC-η-ligand complexes: The x-axis displays a three-letter code of the key residues and the y-axis shows the number of PKC-η-ligand complexes. **B).** Population mode refers to the histogram of fingerprint of the PKC-η-ligand complexes which showing the number of ligands with which each residue interacts. **C).** This display 2D depictions of the ligands. The colour highlights of the ligands correspond to the interactions shown in the barcodes and population modes. Notices that all interactions with a single protein residue will have the same colour in all the display modes.

TABLE

Table S1. Identification of missing regions in crystal structures of cancer drug targets using SEQATOMs. (missing residues were displayed in small case with red colour font with yellow background in the sequence).

PDBID	Sequence
**4A55**	msyyhhhhhhdydipttenlyfqgamdpmpprPSSGELWGIHLMPPRILVECLLPNGMIVTLECLREA<br>TLVTIKHELFREARKYPLHQLLQDETSYIFVSVTQEAEREEFFDETRRLCDLRLFQPFLK<br>VIEPVGNREEKILNREIGFVIGMPVCEFDMVKDPEVQDFRRNILNVCKEAVDLRDLNSP<br>HSRAMYVYPPNVESSPELPKHIYNKLDKGQIIVVIWVIVSPNNDKQKYTLKINHDCVPE<br>QVIAEAIRKKTRSMLLSSEQLKLCVLEYQGKYILKVCGCDEYFLEKYPLSQYKYIRSCIM<br>LGRMPNLMLMAKESLYSQLPIDSFTMPSYSRristatpymngetSTKSLWVINSALRIKILCAT<br>YVNVNIRDIDKIYVRTGIYHGGEPLCDNVNTQRVPCSNPRWNEWLNYDIYIPDLPRAAR<br>LCLSICSVkgrkgakeehCPLAWGNINLFDYTDTLVSGKMALNLWPVPHGLEDLLNPIGVTG<br>SNPNKETPCLELEFDWFSSVVKFPDMSVIEEHANWSVsreagfsyshtglsnrlardnelreNDKEQL<br>RALCTRDPLSEITEQEKDFLWSHRHYCVTIPEILPKLLLSVKWNSRDEVAQMYCLVKD<br>WPPIKPEQAMELLDCNYPDPMVRSFAVRCLEKYLTDDKLSQYLIQLVQVLKYEQYLDN<br>LLVRFLLKKALTNQRIGHFFFWHLKSEMHNKTVSQRFGLLLESYCRACGMYLKHLNR<br>QVEAMEKLINLTDILKQEKKDETQKVQMKFLVEQMRQPDFMDALQGFLSPLNPAHQL<br>GNLRLEECRIMSSAKRPLWLNWENPDIMSELLFQNNEIIFKNGDDLRQDMLTLQIIRIME<br>NIWQNQGLDLRMLPYGCLSIGDCVGLIEVVRNSHTIMQIQCkgglkgalQFNSHTLHQWLK<br>DKNKGEIYDAAIDLFTRSCAGYCVATFILGIGDRHNSNIMVKDDGQLFHIDFGHFLDHK<br>KKKFGYKRERVPFVLTQDFLIVISKGAQEYTKTREFERFQEMCYKAYLAIRQHANLFIN<br>LFSMMLGSGMPELQSFDDIAYIRKTLALDKTEQEALEYFTKQMNDAHHGGWTKMDWI<br>FHTikqhaln
**3TXO**	gpkesskegngigvnssnrlgiDNFEFIRVLGKGSFGKVMLARVKETGDLYAVKVLKKDVILQDD<br>DVECTMTEKRILSLARNHPFLTQLFCCFQTPDRLFFVMEFVNGGDLMFHIQKSRRFDEA<br>RARFYAAEIISALMFLHDKGIIYRDLKLDNVLLDHEGHCKLADFGMCKEGICngvttatfcG<br>TPDYIAPEILQEMLYGPAVDWWAMGVLLYEMLCGHAPFEAENEDDLFEAILNDEVVY<br>PTWLHEDATGILKSFMTKNPTMRLGSLTQGGEHAILRHPFFKEIDWAQLNHRQIEPPFR<br>PRIKSREDVSNFDPDFIKEEPVLXPIDEGHLPMINQDEFRNFEYVSPelqp
**1A3Q**	GPYLVIVEQPKQRGFRFRYGCEGPSHGGLPGASSEKGRKTYPTVKICNYEGPAKIEVDL<br>VTHSDPPRAHAHSLVGKQCSELGICAVSVGPKDMTAQFNNLGVLHVTKKNMMGTMIQ<br>KLQRQRLRSRPQGLTEAEQRELEQEAKELKKVMDLSIVRLRFSAFLRSLPLKPVISQPIH<br>DSKSPGASNLKISRMDKTAGSVRGGDEVYLLCDKVQKDDIEVRFYEDDENGWQAFGD<br>FSPTDVHKQYAIVFRTPPYHKMKIERPVTVFLQLKRKRGGDVSDSKQFTYYP
**1LFD**	MTEYKLVVVGAGGVGKSALTIQLIQNHFVDKYDPTIEDSYRKQVVIDGETCLLDILDTA<br>GQEEYSAMRDQYMRTGEGFLCVFAINNTKSFEDIHQYREQIKRVKDSDDVPMVLVGN<br>KCDLAARTVESRQAQDLARSYGIPYIETSAKTRQGVEDAFYTLVREIRQHK
**2CU1**	GSSGSSGDVRVKFEHRGEKRILQFPRPVKLEDLRSKAKIAFGQSMDLHYTNNELVIPLTT<br>QDDLDKAVELLDRSIHMKSLKILLVINGSTQATNLEPSGPSSG
**3CQW**	GAMDPRVTMNEFEYLKLLGKGTFGKVILVKEKATGRYYAMKILKEVIVAKDEVAHTL<br>TENRVLQNSRHPFLTALKYSFQTHDRLCFVMEYANGGELFFHLSRERVFSEDRARFYG<br>AEIVSALDYLHSEKNVVYRDLKLENLMLDKDGHIKITDFGLCKEGIKDGATMKTFCGT<br>PEYLAPEVLEDNDYGRAVDWWGLGVVMYEMMCGRLPFYNQDHEKLFELILMEEIRFP<br>RTLGPEAKSLLSGLLKKDPKQRLGGGSEDAKEIMQHRFFAGIVWQHVYEKKLSPPFKP<br>QVTSETDTRYFDEEFTAQMITITPPDQDDSMECVDSERRPHFPQFDYSASSTA
**3M06**	sELLQRCESLEKKTATFENIVCVLNREVERVAMTAEACSRQHRLDQDKIEALSSKVQQL<br>ERSIglehhhhhh
**121P**	MTEYKLVVVGAGGVGKSALTIQLIQNHFVDEYDPTIEDSYRKQVVIDGETCLLDILDTA<br>GQEEYSAMRDQYMRTGEGFLCVFAINNTKSFEDIHQYREQIKRVKDSDDVPMVLVGN<br>KCDLAARTVESRQAQDLARSYGIPYIETSAKTRQGVEDAFYTLVREIRQH
	MQSDVRIKFEHNGERRIIAFSRPVKYEDVEHKVTTVFGQPLDLHYMNNELSILLKNQDD<br>LDKAIDILDRSSSMKSLRILLLSQDRNLEHHHHHH

Table S2. The active site residues, pocket volume and surface area of molecular targets were predicted using CASTp server.

Protein	PDB ID	Active site residues	Surface Area (SA)	Volume (SA)
PKC-η	3TXO	Leu361, Gly362, Lys363, Ser365, Phe366, Gly367, Lys368, Val369, Ala382, Lys384, Val385, Ile391, Asp394, Asp396, Cys399, Thr400, Glu403, Leu407, Thr417, Arg428, Phe431, Mrt433, Glu434, Phe435, Val436	843.6	910.5
HRas-P21	121P	Gly12, Gly13, Val14, Gly15, Lys16, Ser17, Ala18, Phe28, Val29, Asp30, Glu31, Tyr32, Thr35, Try40, Asp57, Thr58, Ala59, Gly60, Gln61, Asn116, Lys117, Asp119, Leu120, Ser145, Ala146, Lys147	336.6	224.3
AKT-1	3CQW	Phe161, Gly162, Lys163, Val164, Arg174, Lys179, Leu181, Glu191, His194, Thr195, Glu198, Leu202, Thr211, Met227, Glu228, Tyr229, E234, Met281, Thr312, His354, Lys419, Glu432, Phe438, Phe442	828.6	602.9
Ras	1LFD	Ala211, Gly212, Lys216, Try232, His227, Phe228, Asp230, Tyr232 Thr235, Gln261, Asn285, Lys288, Tyr296, Asn316, Lys317, Asp319, Ala346, Lys347,	951.7	593.8
PI3K-α	4A55	Ala533, Cys53z5, Thr536, Ser541, Glu563, Lys594, Glu596, Gln507, Asp625, Lys627, Ser629, Gln630, Tyr631,Arg662, His665, Phe666, Leu752, Asn756, Met772, Pro778, Trp780, Leu793, Ile800, Lys802, Val850, Val851, Try836, Ile932, Asp933	2703.0	2736.6
MEKK3	2PPH	Asp41, Tyr44, Asn46, Leu49, Ser50, Ile51, Leu52, Lys54, Asn55, Asp57, Asp58, Lys61, Ala62, Ile65, Leu66,	332.6	484.8

		Met72, Gln82, Asn85, His90, His91, His92, His93		
NFκB-P52	1A3Q	Glu92, Asp94, Leu95, Arg103, Ala104, His105, His107, Ser108, Ile117, Gly118, Ile119, Lys153, Leu154, Arg156, Gln157, Arg160, Ser161, Arg193, Ser195, Phe197, Ser206	283.1	327.4
MEKK2b	2CU1	Leu30, Glu31, Arg34, Ser35, Ala37, Lys38, Ser44, Met45, Asp46, Leu47, Leu58, Thr59, Thr60, Gln61, Asp62, Asn87, Ser89, Thr90, Gln91, Ala92, Thr93, Asn94, Leu95, Ser98, Pro100	451.3	609.2
TRAF2	3M06	Glu267, Gln270, Arg271, Lys277, Thr281, Phe282, Thr341, Phe344, Glu400, Thr403, Phe406, Val410, Cys411, Glu521, Glu524, Lys525, Thr527, Glu577, Gln580, Arg581, Ser584, Leu585, Lys587, Lys588, Thr591, Phe592	662.4	1051.0

Table S3. The binding interactions, distance, angle and binding energy of the lead compounds with cancer drug targets.

Dietary Compounds	Protein	Binding Interactions	Distance (Å)	Angle (∠)	Atoms involved in Angle	Binding energy ΔG kcal/mol
Silibinin	H-Ras	Lys117----------CO	2.4	126.0	O-H-CO	-11.6
		Phe28-----------CO	4.0	126.8	N-H-OH	
		Ser17------------CO	3.7	143.1	C-O-OH	
		Asp119----------CO	3.0	164.6	N-H-CO	
		Asp119----------CO	3.2	126.7	O-H-OH	
		Lys16------------CO	3.8	136.7	C-H-OH	
	RAS	Phe228-----------OH	3.9	135.2	C-H-OH	-10.2
		Lys317-----------OC	4.5	117.7	N-H-OH	
		Gly213-----------OH	3.9	107.7	C-O-OH	
		Gly213-----------OH	3.9	152.0	O-O-OH	
		Thr235-----------OC	3.2	171.5	N-H-OH	
		Tyr232-----------OC	4.8	189.9	O-H-OH	
	PKC	Asp440-----------CO	3.6	108.8	C-O-OH	-10.0
		Asp497-----------CO	4.2	121.5	N-H-CO	
		Leu486-----------SO	4.3	135.6	O-H-OH	
		Val436------------SO	3.0	98.7	C-H-OH	
	PI3K	Trp780------------OC	3.4	125.4	N-O-OH	-9.3
		Tyr836------------OC	3.8	122.5	C-O-OH	
		Tyr836------------OC	2.8	109.7	N-H-OH	
		Ile932-------------OC	3.4	113.1	O-H-CO	
		Lys802------------OC	3.7	123.2	C-H-OH	
		Met772------------OH	3.5	95.7	N-H-OH	
		Met772------------OH	4.0	102.7	C-O-OH	
Flavopiridol	PKC	Val369------------ OC	3.1	64.6	N-H-OH	-10.5
		Leu486-------------Cl	4.1	130.9	C-O-NC	
		Asp440-----------NC	3.7	113.1	N-H-OH	
		Asp497-----------OC	3.8	123.2	C-O-OH	
	PI3K	Trp780-----------arene	3.6	90.3	C-O-HO	-9.2
		Tyr836-----------arene	3.6	121.0	N-H-OH	
		Tyr836-----------OC	2.8	120.0	C-H-OH	
		Ile932------------OC	3.6	113.1	O-H-CO	
		Asp933-----------OC	2.8	123.2	N-H-OH	
		Met772------------OC	3.7	95.7	H-H-OH	
	RAS	Asp391---------Cl25	3.5	140.1	N-H-CO	-9.0
		Phe228---------arene	4.0	113.1	C-H-OH	
		Tyr323---------OC	3.7	123.2	N-H-OH	
		Lys317---------OH	3.2	95.7	O-H-OH	
		Ala218---------ON	4.3	102.7	C-O-OH	
Oleandrin	PI3K	Trp780-----------arene	2.9	90.1	N-H-OC	-10.0
		Val850-----------HC	3.5	128.0	C-H-OH	
		Tyr836-----------arene	3.1	108.5	N-H-CO	
		Ile932------------OC	2.9	85.5	O-H-OH	
		Ile932------------OC	3.6	111.5	N-H-OH	

	RAS	Phe228-----------OH	3.8	128.5	H-N-OH	-9.7
		Ala218-----------OC	4.4	132.1	H-N-OH	
		Ser217-----------OC	4.1	132.4	C-O-OH	
	PKC	Leu486-----------OH	3.6	111.4	C-O-HC	-9.2
		Val436------------OC	3.0	102.2	O-C-OC	
		Ala382------------OC	3.4	117.5	N-H-OH	
		Val369------------HC	3.8	149.3	O-C-OC	
α-Boswellic acid	PI3K	Trp780----------- Arene	3.3	124.9	N-H-OH	-10.2
		Ile932-------------CO	3.7	101.2	C-O-OH	
		Met772-----------OH	3.6	164.4	N-H-OH	
		Lys802-----------HC	3.4	156.7	N-C-OH	
	RAS	Lys317------------CH	3.4	112.4	C-H-OC	-9.1
		Phe228------------CH	3.5	131.6	O-C-OH	
		Ser217-------------OH	3.2	98.1	C-O-OC	
		Tyr232-------------OH	3.7	103.1	N-H-OH	
β-Boswellic acid	PI3K	Trp780------------Arene	3.5	109.8	N-H-OH	-9.0
		Tyr836-------------CH	3.9	103.0	C-O-OH	
		Met772-------------OH	4.5	116.3	C-N-OH	
Triterpenoid	PI3K	Trp780------------ OC	4.3	138.3	C-N-OC	-10.6
		Val851-------------OC	3.8	103.1	H-C-OH	
		Tyr836-------------CH	2.8	92.3	C-O-HO	
Guggulesterone	PI3K	Tyr836-------------CH	3.8	113.1	N-H-OH	-10.0
		Trp780------------ OC	4.0	123.2	H-O-CO	
		Ile800------------- OC	3.3	95.7	C-H-OH	
		Met772----------- OC	3.9	102.7	N-C-OC	
Ursolic acid	PI3K	Tyr836------------CH	2.9	106.2	C-O-OH	-9.0
		Trp780----------- OC	3.4	109.6	N-H-OC	
		Met772---------- OC	4.5	162.8	N-C-NH	
	PKC	Val369------------OC	3.2	122.1	O-H-CH	-10.4
		Leu486------------HC	4.2	118.1	C-N-OH	
		Asp497------------OC	2.9	123.5	N-H-OC	
Oleanolic acid	PI3K	Ile932--------------OC	3.6	84.0	N-C-OH	-9.0
		Trp780-------------HC	3.4	113.1	O-H-CO	
		Asp933------------ HC	3.9	123.2	N-O-OH	
		Asp933------------ HC	3.3	95.7	C-H-OH	
Emodin	PKC	Lys834-------------OC	4.1	122.4	O-H-OH	-9.1
		Leu486-------------OH	3.0	113.1	C-H-CO	
		Val436-------------OC	4.0	123.2	N-H-CH	
		Val369-------------OC	3.7	95.7	N-H-OH	
		Val369-------------OC	3.9	102.7	C-O-OH	
Ellagic acid	PKC	Lys834------------OC	5.0	144.6	C-O-HO	-9.4
		Asp497------------OH	3.2	113.9	N-H-OC	
		Val436-------------OC	3.8	141.0	N-H-HO	
		Ala382-------------OC	3.5	161.5	C-O-HO	
		Val369-------------OC	4.3	143.6	N-H-CO	

Table S4. 2D structures, binding energy, binding affinity, MM/GBVI, efficiency and Lipinski rule of the best lead molecules of PI3K-α.

S. No	Structure	Binding energy (kcal/mol)	Binding affinity (pKi)	MM/GBVI (kcal/mol)	Efficiency	MW	logP	TPSA	Don	Acc
1	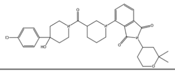	-10.0	5.7	-21.0	0.141	580.1	4.9	90.0	1	5
2	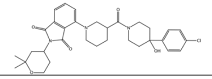	-9.2	4.6	-14.8	0.113	580.1	4.9	90.3	1	5
3	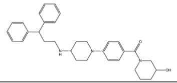	-9.0	6.3	-20.3	0.171	497.6	5.0	55.8	2	3
4	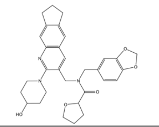	-8.9	5.2	-14.0	0.133	529.6	4.6	84.3	1	6
5	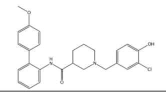	-8.9	6.2	-10.7	0.195	450.9	5.8	61.8	2	4
6	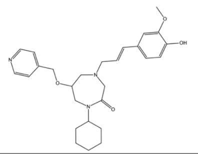	-8.7	7.3	-23.6	0.216	465.5	4.1	75.1	1	6
7	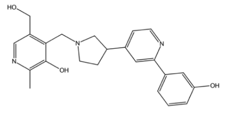	-8.6	5.5	-15.4	0.190	391.4	3.8	89.7	3	6

8	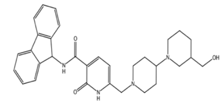	-8.5	5.4	-20.4	0.144	512.6	3.0	84.9	3	5
9	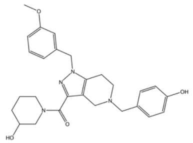	-8.5	5.7	-16.6	0.164	476.5	3.6	91.0	2	6
10	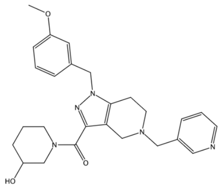	-8.5	7.2	-20.9	0.194	461.5	3.2	83.7	1	6

Table S5. 2D structures, binding energy, binding affinity, MM/GBVI, efficiency and Lipinski rule of five of the best lead molecules of PKC-η.

S. No	Structure	Binding energy (kcal/mol)	Binding affinity (pKi)	MM/GBVI (kcal/mol)	Efficiency	MW	logP	TPSA	Don	Acc
1	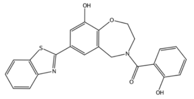	-11.9	8.7	-31.8	0.292	418	4.6	82.8	2	5
2	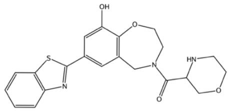	-10.5	9.6	-11.0	0.333	411	2.6	83.9	2	6
3	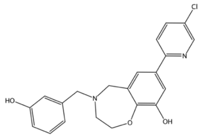	-10.4	8.7	-31.8	0.292	418	4.6	82.8	2	5
4	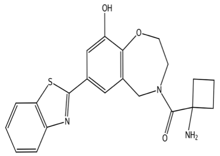	-9.9	10.0	-21.0	0.358	395	3.5	88.6	2	5
5	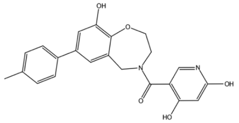	-9.4	9.1	-24.7	0.317	392	3.4	103.1	3	6

6	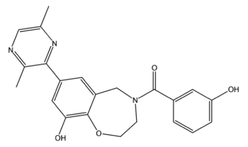	-9.4	7.9	-22.1	0.274	391	3.4	95.7	2	6
7	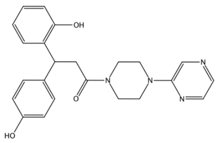	-9.4	10.6	-27.2	0.356	404	2.7	89.7	2	5
8	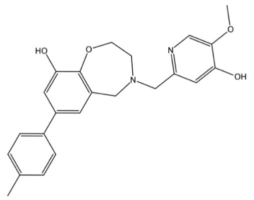	-9.3	8.0	-21.1	0.277	392	4.4	75.0	2	6
9	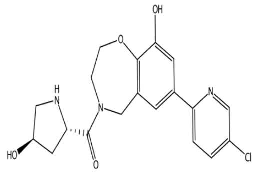	-9.3	8.6	-21.6	0.320	389	1.8	94.9	3	6
10		-9.2	6.7	-29.0	0.234	399	3.8	62.1	2	5

	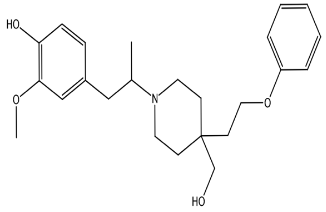									
11	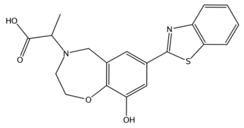	-9.1	9.0	-14.6	0.349	370.4	3.6	82.8	2	6
12	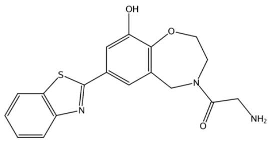	-8.9	8.9	-17.7	0.357	355.4	2.6	88.6	2	5
13	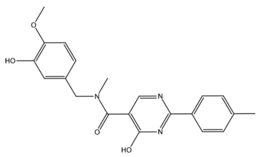	-8.9	8.8	-15.2	0.316	379	3.4	95.7	2	6
